# Telmisartan, an anti-hypertensive drug, impedes JEV infection, possibly via the AT1/PPARγ axis

**DOI:** 10.1128/spectrum.03003-24

**Published:** 2025-08-14

**Authors:** Ankita Datey, Sanchari Chatterjee, Soumyajit Ghosh, P Sanjai Kumar, Saikat De, Udvas Ghorai, Debasish Hota, Bharat Bhusan Subudhi, Soma Chattopadhyay

**Affiliations:** 1 BRIC- Institute of Life Sciences 29722, Bhubaneswar, Odisha, India; 2 School of Biotechnology, Kalinga Institute of Industrial Technology 563308 , Bhubaneswar, India; 3 Viral Mutation Section, HIV dynamics and Replication Program, Center for Cancer Research, National Cancer Institute at Frederick 437329, Frederick, Maryland, USA; 4 Regional Centre for Biotechnology 214253, Faridabad, India; 5 Division of Neonatology and Newborn Nursery, University of Wisconsin—Madison, Madison, Wisconsin, USA; 6 Scripps Research, La Jolla, California, USA; 7 Department of Pharmacology, AIIMS 410775, Bhubaneswar, India; 8 School of Pharmaceutical Sciences, Siksa 'O' Anusandhan Deemed to be University 177142 , Bhubaneswar, Odisha, India; National University of Singapore, Singapore, Singapore

**Keywords:** JEV, telmisartan, AT1, PPARγ, BHK-21 cells, Huh7 cells

## Abstract

Japanese encephalitis, caused by Japanese encephalitis virus (JEV), is a vector-borne disease with no specific therapeutics available yet. Binding of angiotensin II (Ang II) to angiotensin II type 1 (AT1) receptor induces the release of inflammatory cytokines associated with viral encephalitis. Accordingly, Ang II receptor blockers (ARBs) have been proposed to manage encephalitis. Since telmisartan (TM, antagonist of AT1 and agonist of PPARγ) has relatively better brain access than other ARBs, this investigation aims to evaluate its anti-JEV efficacy *in vitro* and *in vivo*. TM reduced JEV titer, RNA, and protein (NS3) significantly in the BHK-21 cells with an IC_50_ of 24.68 µM and a CC_50_ of >350 µM (Selectivity Index >14.18), indicating its potential for repurposing against JEV. The anti-JEV efficacy of TM was further observed in other physiologically relevant cells. Interestingly, the viral load was reduced significantly in pre-, co-, and post-treatment conditions of TM. In the presence of GW (PPARγ antagonist) and AG (AT1 agonist), viral infection was increased remarkably, while AT1 was upregulated and PPARγ was downregulated. TM treatment reversed these levels during infection. In addition, siRNA knockdowns of AT1 and PPARγ showed an insignificant change in infection upon TM treatment. Furthermore, reduction of inflammatory markers like p-IRF-3, COX-2, and p-NF-κB was observed after TM treatment in RAW264.7 cells, suggesting its immunomodulation through the AT1/PPARγ axis. Finally, the anti-JEV potential of TM was validated in a mouse model through the reduction of disease score, viral protein, and histological changes. Thus, the preclinical efficacy of TM suggests its suitability for repurposing against JEV.

**IMPORTANCE:**

Japanese encephalitis, caused by JEV, is a vector-borne disease for which no specific therapeutics are yet available. Binding of Ang II to the AT1 receptor induces the release of inflammatory cytokines associated with viral encephalitis. Accordingly, telmisartan, which is an antagonist of AT1 and an agonist of PPARγ with better brain access, was evaluated for its anti-JEV efficacy *in vitro* and *in vivo* in the current study. Telmisartan demonstrated significant reduction in JEV titer, RNA, and protein levels in different treatment conditions. This treatment reversed the levels of AT1 and PPARγ as well as inflammatory markers, p-IRF-3, COX-2, and p-NF-κB during JEV infection, suggesting its immunomodulatory effect through the AT1/PPARγ axis. Finally, the anti-JEV potential of telmisartan was validated in a mouse model through the reduction of disease score, viral protein, and histological changes. Hence, the preclinical efficacy of telmisartan suggests its suitability for repurposing against JEV.

## INTRODUCTION

Japanese encephalitis virus (JEV), a mosquito-borne virus, belongs to the family *Flaviviridae*. It comprises single-stranded positive-sense RNA, and the size of the genome is around 11 kb. Its genome is divided into three structural proteins, namely capsid (C), pre-membrane (prM), and envelope (E), and seven non-structural proteins (NS1, NS2A, NS2B, NS3, NS4A, NS4B, and NS5) (1). It primarily causes encephalitis, affecting children up to 14 years of age, whereas adults develop immunity after infection (2). JEV is transmitted by *Culex* species of mosquitoes, mainly *Culex vishnuii* and *Culex tritaeniorhynchus*. Pigs act as an amplifier host, whereas bats, along with Ardeid birds (herons and egrets), act as a primary reservoir host for JEV. With prolonged viremia, mosquito vectors transmit JEV to humans who are considered dead-end hosts. The common signs and symptoms include fever, nausea, headache, movement disorders, whereas severe conditions result in permanent neurological manifestations, which often lead to mortality (3). Although incidences of encephalitis are limited to approximately 1% of the JEV-infected population, higher rates (20%–30%) of mortality are associated with individuals developing JE. Furthermore, 50% of patients who survive JE develop neuropsychiatric sequelae (4). Globally, 30,000–50,000 incidences of JEV are reported each year, mostly from 24 countries in South-East Asia and the Western Pacific, where it is endemic (3). Faraway countries, including Australia, have also been identified as risk areas by the Centers for Disease Control (CDC), USA.

Vaccines are available for JEV. However, high cost, multiple doses, emerging genotypes, and poorly coordinated JEV control programs are some of the challenges to its effectiveness in preventing JE (5). Furthermore, the enzootic cycle of JEV makes it difficult to completely eradicate. Thus, there is a need for its therapeutic management. Although this has drawn attention for antiviral research, no specific therapy is available for application. Drug repurposing is a better alternative to new drug development to minimize cost and time for developing an antiviral strategy. Accordingly, several drugs such as minocycline, interferon, ribavirin, immunoglobulin, dexamethasone, and acyclovir have been investigated against JEV (6). Out of these, only minocycline showed a statistically significant decrease. However, to date, no specific antiviral drug is available against JEV, which necessitates exploring alternative approaches (4).

Considering that encephalitis is the major reason for mortality and morbidity associated with JEV infection, it is worthwhile to explore drugs capable of reducing neuroinflammation for anti-JEV therapy. Because of the involvement of the local renin–angiotensin system (RAS) in neuroinflammation, inhibiting RAS has been documented as a strategy to minimize encephalitis (7). Binding of angiotensin II (Ang II) to angiotensin II type 1 (AT1) receptor is known to induce release of inflammatory cytokines associated with viral encephalitis (8). Accordingly, angiotensin II receptor blockers (ARBs) have been proposed to manage encephalitis. However, the majority of the approved ARBs have poor access to the brain because of their inability to cross the blood-brain barrier (BBB). Nevertheless, increasing their brain access has been shown to enhance protection against neuroinflammation (9). Unlike most of these ARBs, telmisartan (TM) has relatively better brain access and selectively blocks AT1R activation (proinflammatory axis) without hindering the signaling cascade of AT2R (anti-inflammatory axis) (10). Furthermore, it is also a partial agonist of peroxisome proliferator-activated receptor gamma (PPAR)γ. Downregulation of PPARγ is an important marker of viral infection-induced inflammation in the brain (11). Hence, TM can be useful to manage encephalitis associated with JEV. Interestingly, our group has earlier shown antiviral properties of TM against Chikungunya virus (CHIKV), partly mediated through modulation of PPARγ and AT1 (12). Few studies have also shown antiviral properties of AT1 blockers against Venezuelan equine encephalitis virus (VEEV) and flavivirus infections like Dengue (13, 14). Since there are no such reports available against JEV till now, the current study aims to evaluate the anti-viral efficacy of TM against JEV *in vitro* and *in vivo*.

It was observed that TM was able to reduce the viral titer by 90% *in vitro*. To better understand the mechanism of action, GW (PPARγ antagonist) and AG (AT1 agonist) were used. In the presence of GW and AG, viral infection was enhanced, suggesting a role of AT1/PPARγ in JEV infection. Using siRNA against AT1 and PPARγ further confirmed that the mode of action of TM might be through the AT1/PPARγ axis. Furthermore, the detailed mechanism of action of TM revealed downregulation of p-IRF-3 (phospho-interferon regulatory factor 3), COX-2 (cyclooxygenase-2), and p-NF-κB (phospho-nuclear factor kappa-light-chain-enhancer of activated B cells) responsible for inflammation. It was further validated by *in vivo* studies in Balb/c mice model where the JEV-RNA and NS3 protein were reduced to approximately 40% and 60% in TM-treated mice brain as compared to the infection. Thus, the above findings reveal that TM might be repurposed efficiently to restrict JEV infection.

## RESULTS

### Telmisartan hinders JEV infection *in vitro*

**Fig 1:**
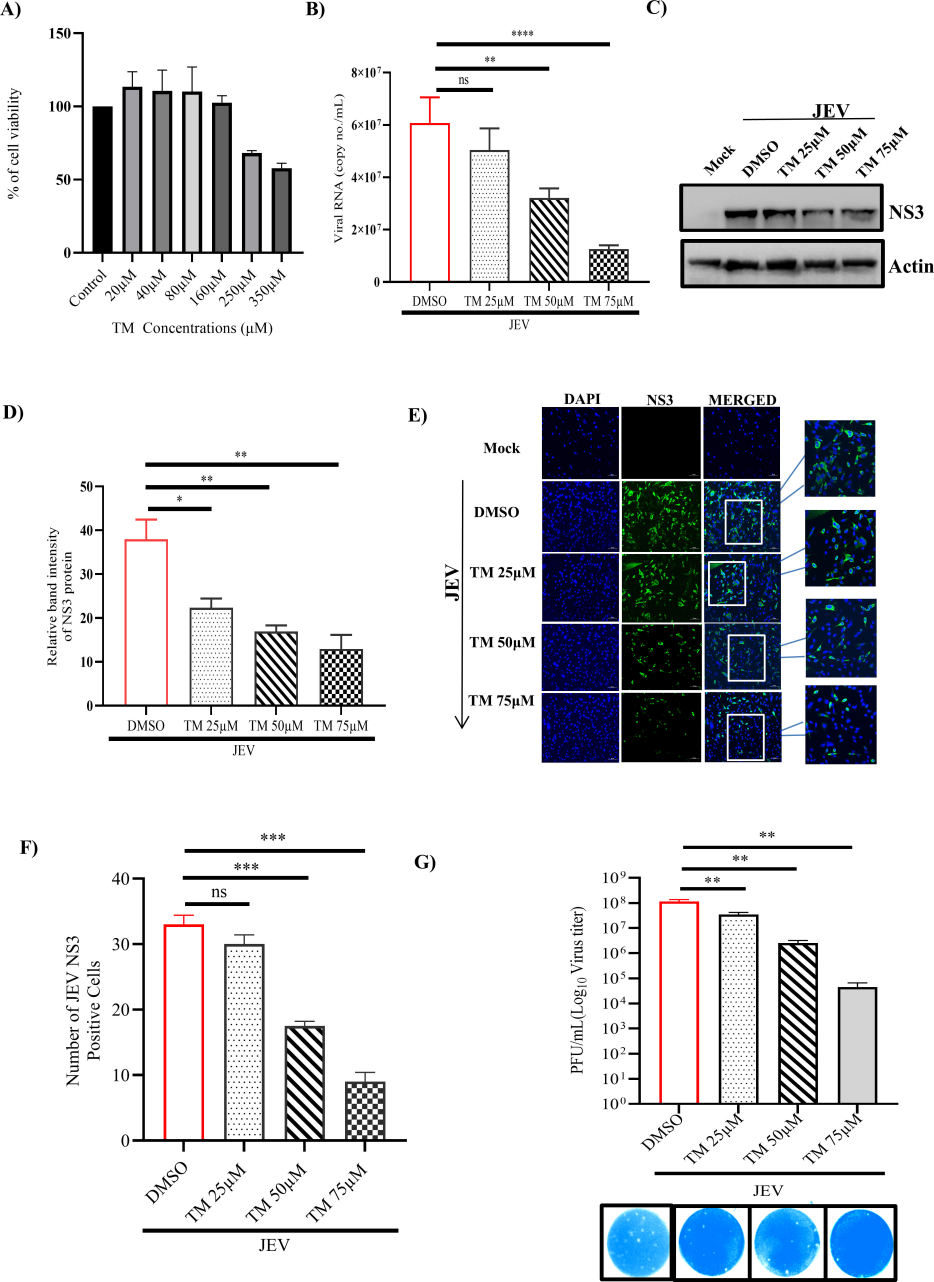
TM possesses anti-JEV activity: BHK-21 cells were infected with JEV at an MOI of 0.1, and TM was administered at varying concentrations (25, 50, and 75 µM) post-infection. The supernatant and pellets were harvested after 24 hpi and subjected to qRT-PCR and Western blot, respectively. (**A**) Bar diagram showing the viability of cells in the presence of TM. (**B**) Bar diagram representing viral RNA copy no./mL with increasing TM concentration. (**C**) Western blot image depicting the level of the JEV-NS3 protein in samples treated with varying concentrations of TM. (**D**) Bar diagram showing relative band intensities of the JEV-NS3 protein. (**E**) Confocal image showing mock, infected, and TM-treated cells, stained with NS3 antibody, and nuclei were counterstained with DAPI (scale bar 100 µM). (**F**) Bar diagram representing the percentage of JEV-NS3-positive cells. (**G**) Bar diagram showing the viral titers (PFU/mL) of different samples. The lower panel shows the images of plaque assay plates. The analysis was carried out using the one-way ANOVA test. *P ≤ 0.05; **P ≤ 0.01; ***P ≤ 0.001; and ****P ≤ 0.0001 were considered statistically significant and ns, not significant (*n* = 3).

**Fig 2:**
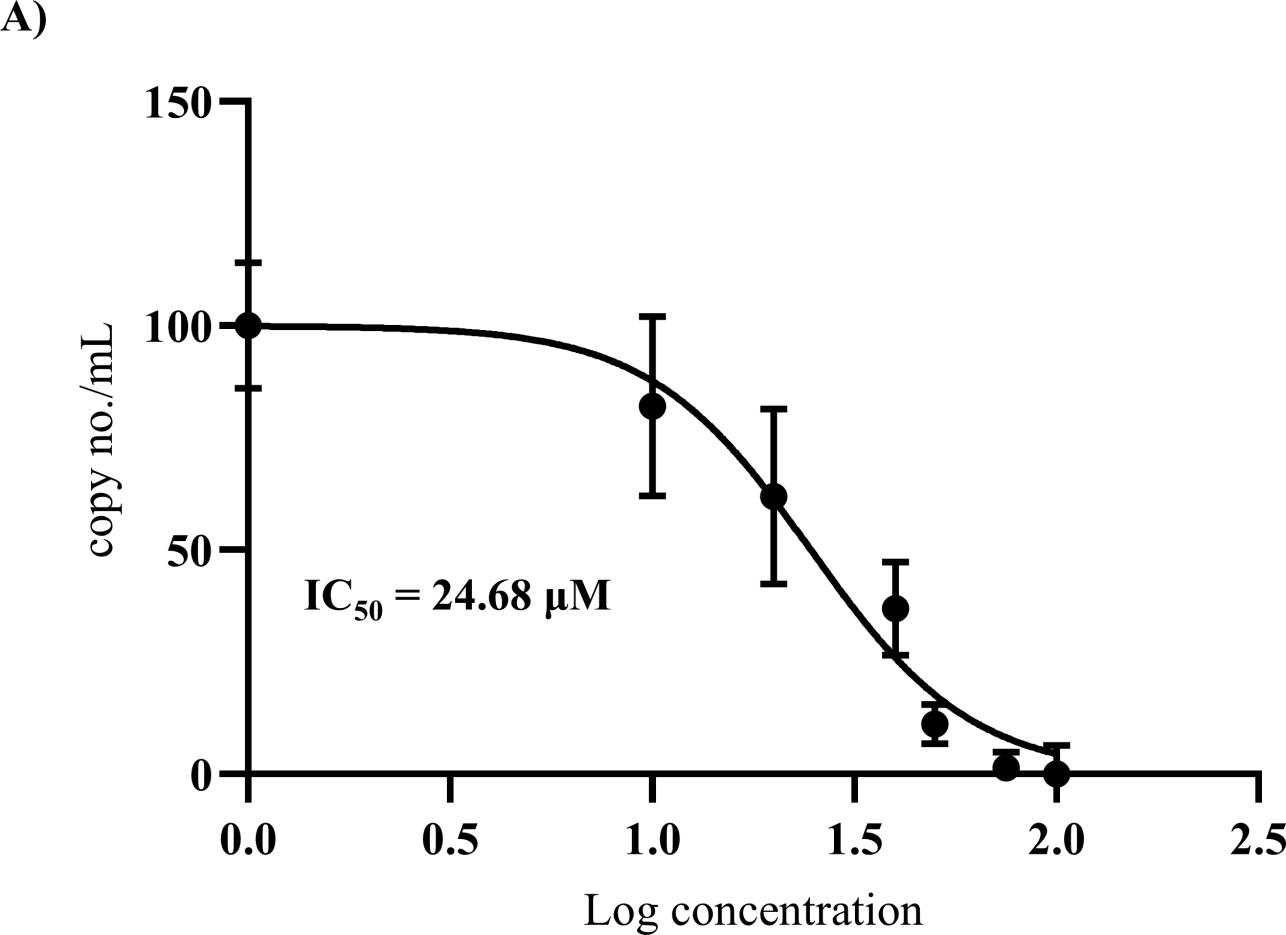
Determining the IC_50_ value of TM: For calculating the IC_50_ value of TM, BHK-21 cells were infected with JEV (MOI 0.1), and different concentrations of TM (10, 20, 30, 40, 50, 75, 100, 125, and 150 µM) were added post-infection. The supernatant was collected, and qRT-PCR was performed to determine the viral RNA as copy no./mL. (**A**) Graph depicting IC_50_ of TM, where the X-axis represents the logarithmic value of varying TM concentrations and the Y-axis represents the percentage of viral RNA in terms of copy no./mL.

To understand the cytotoxicity of TM in BHK-21 (Baby Hamster Kidney-21) and Huh7 (Human Hepatocarcinoma) cells, MTT (3-(4,5-dimethylthiazol-2-yl)−2,5-diphenyltetrazolium bromide) assay was performed with different concentrations of this molecule, and 50% cytotoxicity (CC_50_) was determined. It was observed that the drug was non-cytotoxic till 350 and 100 µM concentrations in BHK-21 and Huh7 cells, respectively (Fig. 1A and Fig. S1J). To determine the antiviral efficacy of TM, BHK-21 cells were infected with JEV at a multiplicity of infection (MOI) of 0.1, and TM was administered post-infection at varying concentrations (25, 50, and 75 µM). The cell culture supernatant and cells were harvested at 24 hours post-infection (hpi). It was observed that there was a significant dose-dependent reduction of 25%, 50%, and 75% in viral copy no/mL and 36%, 52%, and 70% reduction in JEV-NS3 (JEV-Nonstructural protein 3) protein level, approximately (Fig. 1B, C, and D). The data were further supported by the confocal analysis (Fig. 1E and F). Furthermore, the viral titer showed remarkable reductions of 80%, 90%, and 95% with increasing concentrations (25, 50, and 75 µM) of TM, respectively (Fig. 1G). The IC_50_ (inhibitory concentration 50) of TM was found to be 24.68 µM (Fig. 2A), and the selectivity index was observed to be 14.18 (Supplementary Table S2). Furthermore, to investigate the antiviral activity of JEV in physiologically relevant cell lines such as RAW264.7 and SH-SY5Y cells, JEV infection was carried out at an MOI of 5 and 0.1, respectively. It was observed that JEV-E (Envelope) gene expression was decreased up to 60%, 70%, and 80% at 25, 50, and 75 µM concentrations of TM, respectively, in RAW264.7 cells through qRT-PCR. Whereas up to 10%, 25%, and 75% reduction was observed at 25, 50, and 75 µM concentrations of TM in SH-SY5Y cells, respectively (Fig. S1A and D). Similarly, INF-β (interferon-β) gene expression and iNOS (inducible nitric oxide synthase) levels in RAW264.7 cells were also downregulated remarkably with TM treatment (Fig. S1B and C). Moreover, expression of the Cas3 (Caspase3) gene in SH-SY5Y cells was also downregulated significantly in TM-treated samples (Fig. S1E). Also, viral load was reduced to 55% and 65.5%, 80% and 75% at 81.5% at 50 and 75 µM concentrations of TM in HEK293 (Human Embryonic Kidney 293) and Huh7 cells, respectively (Fig. S1F and G). The JEV titer was reduced to 60%, 78% and 56%, 85% at 50 and 75 µM concentrations of TM in HEK293 and Huh7 cells, respectively (Fig. S1H and I). Thus, the data suggest that TM efficiently inhibits the JEV infection *in vitro* in various cell lines and also downregulates levels of inflammatory cytokines and apoptotic markers.

### Pre-, co-, and post-treatment of TM can abrogate JEV infection

**Fig 3:**
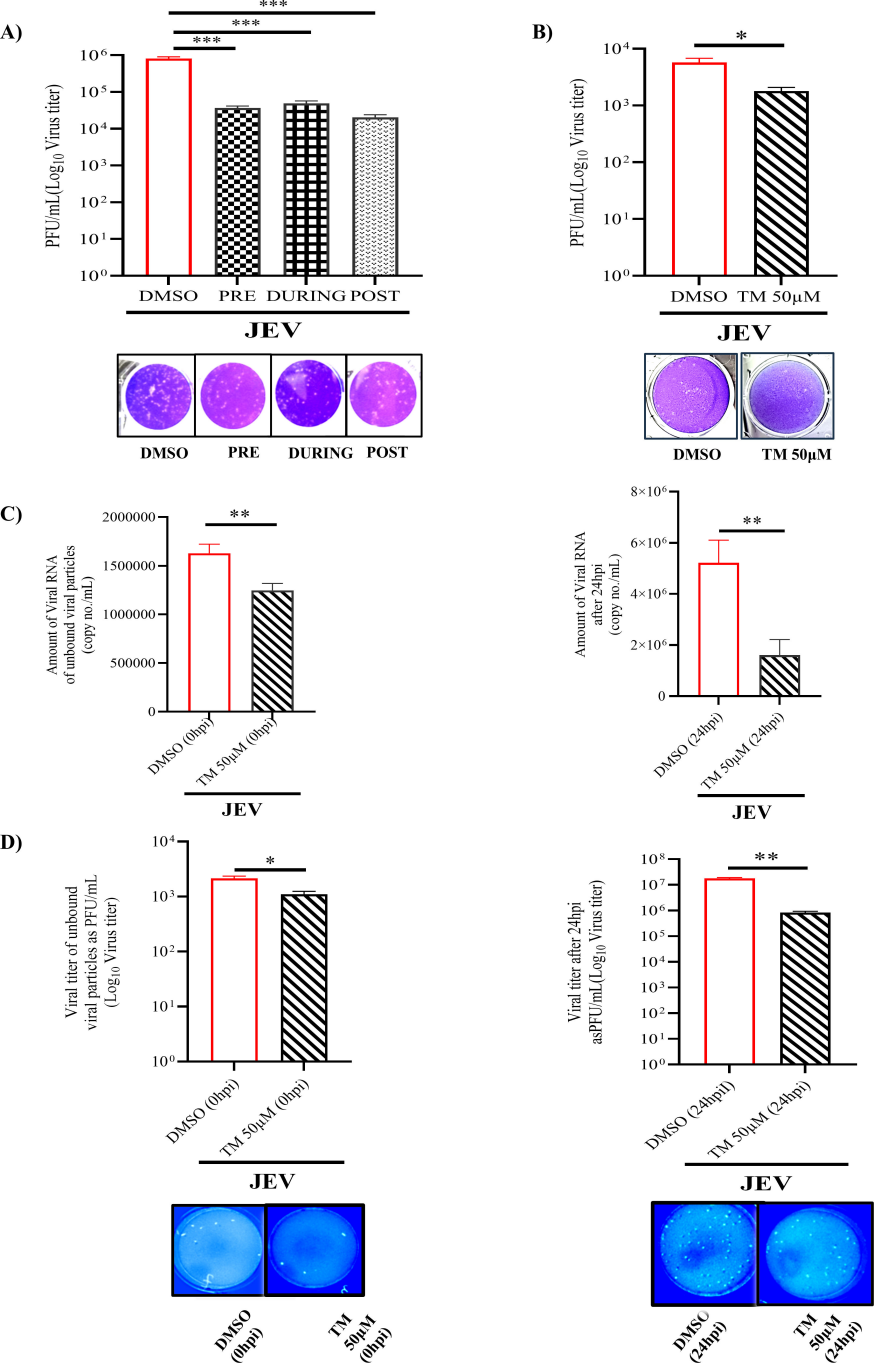
TM inhibits JEV infection in pre-, co-, and post-treatment: BHK-21 cells were treated with TM (50 µM) for 3 hours prior to infection (MOI 0.1) in pre-condition; in the during condition, TM was given along with virus, whereas in post-condition, TM was administered post-infection. The supernatant was collected after 24 hpi, and a plaque assay was performed. (**A**) Bar diagram representing viral titers in all three conditions. The lower panel represents the images of plaque assay plates. (**B**) Bar diagram representing the virucidal activity of TM in the absence or presence of the drug. The lower region shows the image of plaque assay plates. (**C**) Bar diagram representing the viral RNA copy no./mL in different samples (unbound virus particles; left panel, whereas viral particles formed after 24 hpi; right panel). (**D**) Bar diagram showing the viral titers in various samples (unbound virus particles forming plaques; left panel, whereas viral particles collected at 24 hpi generating plaques; right panel). The lower panel depicts the images of plaque assay plates. The data shown are the repetition of three individual experiments with standard mean represented as ±SD. The one-way ANOVA and unpaired two-tailed Student’s *t* test were performed for all the experiments. *P ≤ 0.05; **P ≤ 0.01; ***P ≤ 0.001 were considered statistically significant.

To identify at which stage of the viral life cycle TM (50 µM) can inhibit JEV infection, the drug was administered in pre-, co-, and post-infection conditions as mentioned above. It was observed that TM efficiently reduced the viral titer in all three conditions to 50%, 40%, and 70% (approx), respectively (Fig. 3A). Furthermore, to find out whether TM has a direct impact on JEV viral particles, virus inactivation or virucidal assay was performed. There was significant inhibition (approximately 30%) in viral titer in drug-treated samples as that of infection control (Fig. 3B). Thus, TM might directly impair JEV particles, which may interfere with attachment and/or entry. For a better understanding of the effect of TM in entry or attachment, infection was carried out with JEV in the presence or absence of TM. After the infection was over, unbound virus particles as well as the new particles formed after 24 hpi were collected, and the viral copy no./mL and viral titers (PFU/mL) were determined. Interestingly, it was observed that viral copy no. as well as the titer of unbound particles in the presence of TM was less as compared to unbound virus particles in the absence of TM. Furthermore, the new viral particles formed after 24 hpi were remarkably reduced in the presence of TM (viral copy no and titer) as that of infection control (Fig. 3C and D). Together, the data suggest that TM interferes with JEV infection in different stages of its life cycle.

### TM modulates JEV infection through the AT1/PPARγ pathway

**Fig 4:**
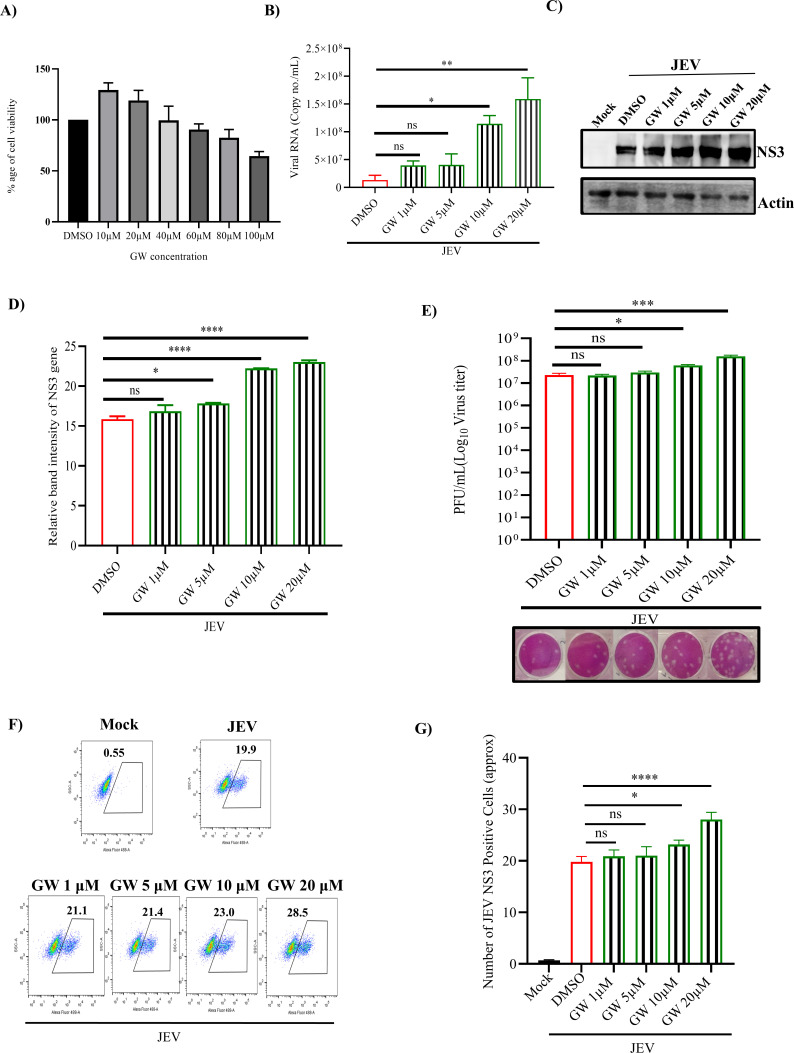
GW (PPARγ antagonist) enhances viral load: JEV infection was carried out in GW (1, 5, 10, and 20 µM) pretreated (3 hours) BHK-21 cells, and GW was administered again after infection. The supernatant and pellets were collected at 24 hpi. (**A**) Bar graph representing the cytotoxicity of GW. (**B**) Bar diagram representing the viral RNA in terms of copy no./mL in different samples. (**C**) Western blot image representing the JEV-NS3 protein level in different samples. Actin was used as a loading control. (**D**) Bar diagram depicting the relative band intensity of JEV-NS3 protein. (**E**) A bar diagram represents the viral plaques in various samples. The lower panel represents the plaque assay plate image. (**F and G**) Dot plot analysis and bar diagram showing the number of JEV-NS3-positive BHK-21 cells. All the experiments were carried out in triplicate, and calculation was done using the one-way ANOVA test. *P ≤ 0.05; **P ≤ 0.01; and ****P ≤ 0.0001 were considered statistically significant. ns, not significant

**Fig 5:**
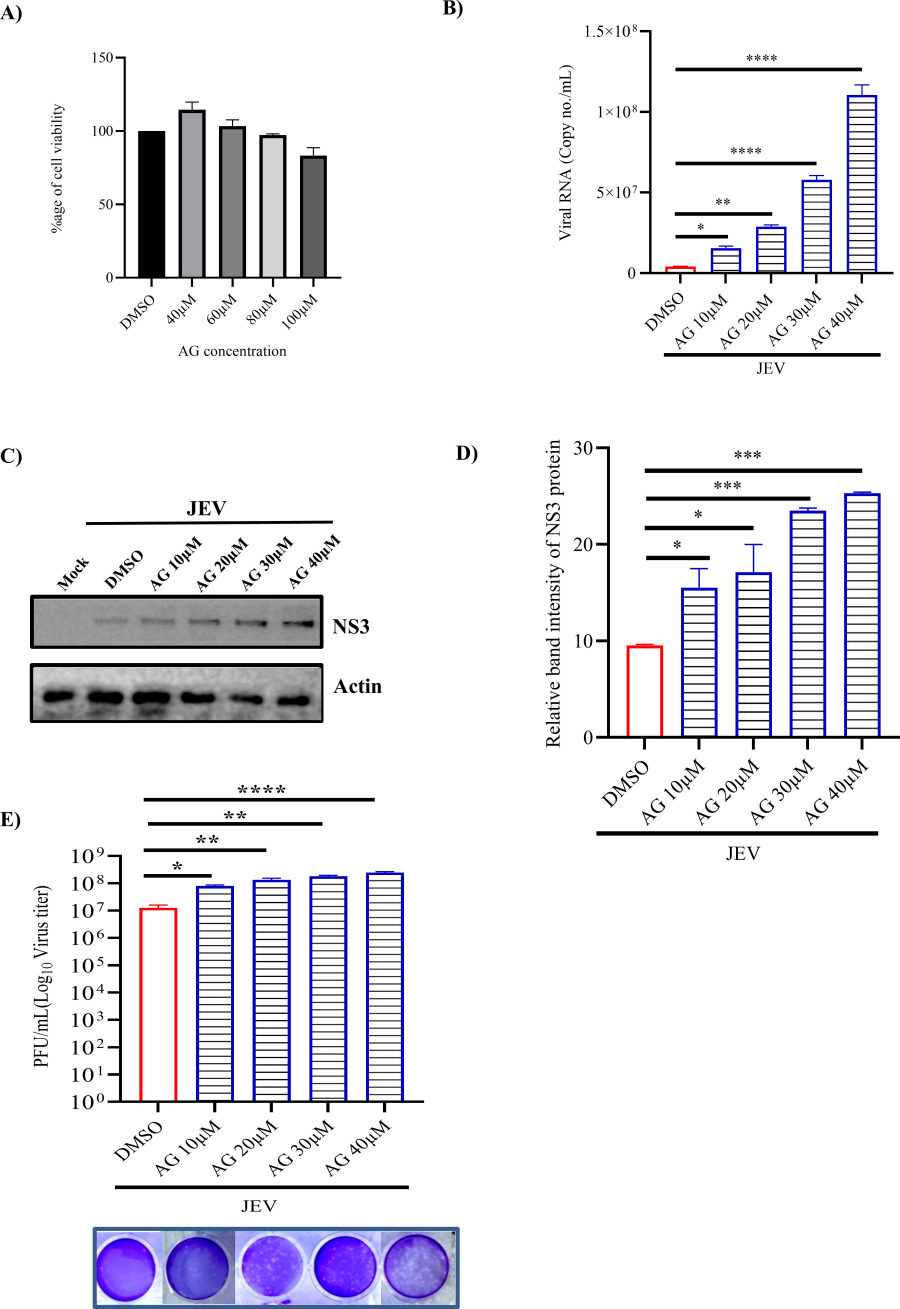
AG, an AT1 agonist, augments viral infection: The BHK-21 cells were pretreated for 3 hours with varying AG concentrations (10, 20, 30, and 40 µM). Later, JEV infection (MOI 0.1) was given to the cells, and AG was administered again post-infection. After 24 hpi, supernatant and pellet were harvested. (**A**) Bar graph showing the percentage of viable cells. (**B**) Bar graph indicating the viral RNA in terms of copy no./mL in various samples. (**C**) Western Blot image showing the band intensity of the JEV-NS3 protein in various samples. Actin was used as a loading control. (**D**) Bar diagram depicting the relative band intensity of the JEV-NS3 protein. (**E**) Bar diagram representing the viral titers as PFU/mL in various samples. The lower panel shows the images of the plaque assay plate. All the statistical analysis was carried out using the one-way ANOVA test. Data were representative of three independent experiments. *P ≤ 0.05; **P ≤ 0.01; ***P ≤ 0.001 were considered statistically significant.

As TM is the antagonist of AT1 and agonist of PPARγ, an antagonist of PPARγ (GW) and an agonist of AT1 receptor (AG) were utilized to understand the mechanism of action of TM in JEV infection. First, their cytotoxicity was determined. GW and AG were found to be non-toxic till 80 µM and 100 µM concentrations, respectively (Fig. 4A and 5A). The BHK-21 cells were pretreated for 3 hours with increasing concentrations of GW (1, 5, 10, and 20 µM) or AG (10, 20, 30, and 40 µM). Post-infection, again, the compounds were added, and samples (supernatant and cells) were harvested after 24 hpi. It was observed that the viral copy number, JEV-NS3 protein level, and viral titer were significantly increased with increasing concentrations of GW and AG (Fig. 4B through E, 5B through E). Similar observations were also noticed by the confocal microscopy (Fig. S2 A through D). Furthermore, the FACS data revealed a significant increase in the expression of the JEV-NS3 protein with increased concentrations of GW (Fig. 4F and G). The higher dose of the inhibitor (GW) also followed a similar pattern (Fig. S2E and F).

**Fig 6:**
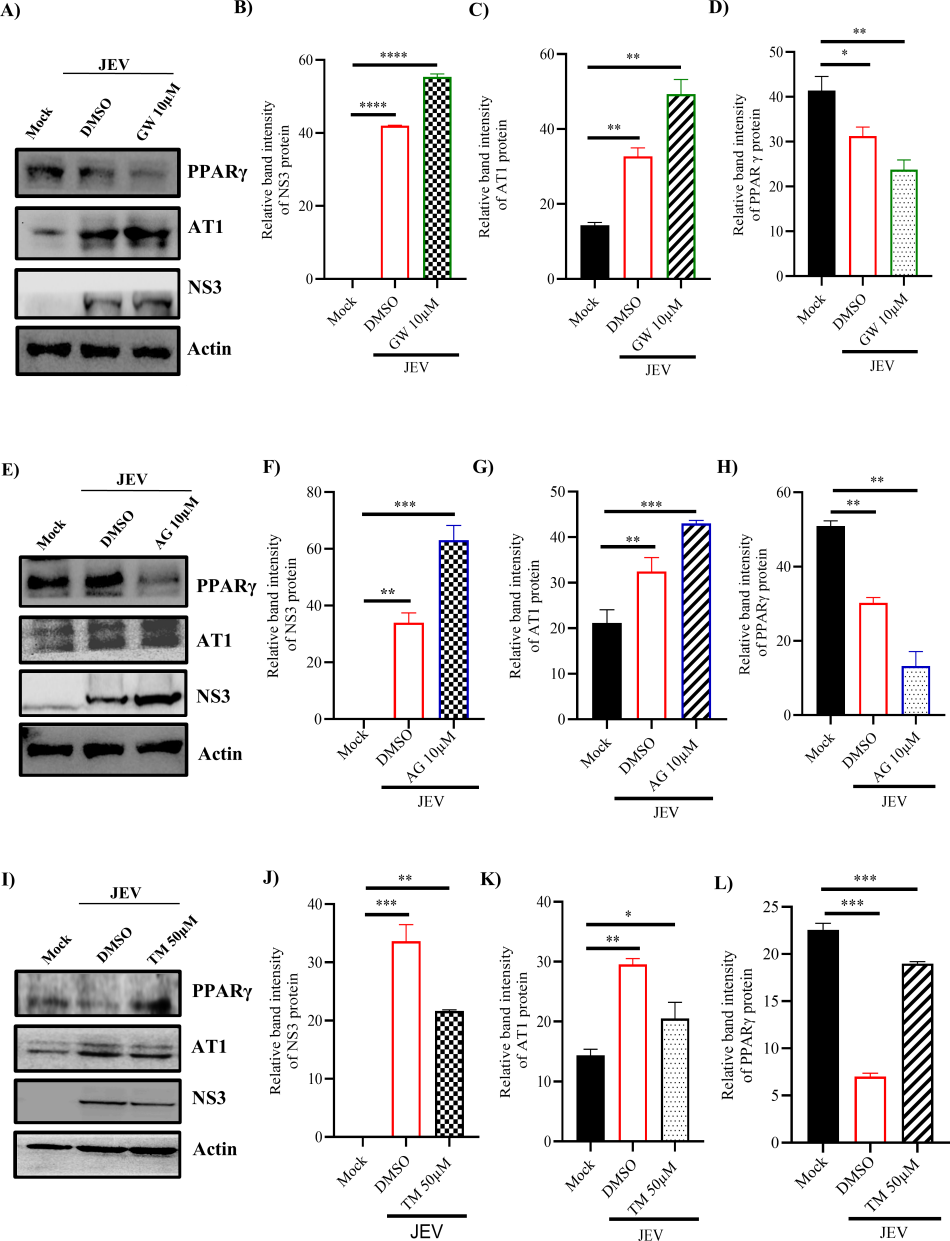
TM reduces JEV infection via AT1/PPARγ pathways**:** BHK-21 cells were pretreated for 3 hours with GW (10 µM), AG (10 µM), and TM (50 µM) separately prior to JEV infection (MOI 0.1). AG, GW, and TM were added post-infection also, and cell pellets were harvested at 24 hpi**. **(**A**) Western blot image depicting expression of JEV-NS3, AT1, and PPARγ proteins in mock, infected, and infected with GW-treated cells. (**B–D**) Bar diagram depicting relative band intensities of JEV-NS3, AT1, and PPARγ proteins in different samples. (**E**) Western blot image showing JEV-NS3, AT1, and PPARγ protein expressions in mock, infected, and infected with AG-treated BHK-21 cells. (**F–H**) Bar diagrams representing the relative band intensities of JEV-NS3, AT1, and PPARγ in different samples. (**I**) Western blot image representing the change in expressions of JEV-NS3, AT1, and PPARγ in mock, infected, and TM-treated samples. (**J–L**) Bar diagrams depicting relative band intensities of JEV-NS3, AT1, and PPARγ in various samples. The one-way ANOVA test analysis was performed; *P ≤ 0.05; **P ≤ 0.01; ***P ≤ 0.001; and ****P ≤ 0.0001 were considered statistically significant.

Previous report from our group suggests that TM alters AT1 and PPARγ expressions (12). Therefore, BHK-21 cells were pre-treated for 3 hours as well as post-treated with GW (10 µM) or AG (10 µM) or TM (50 µM) after infection (MOI 0.1). The cells were harvested at 24 hpi and processed for Western blot. It was observed that the JEV-NS3 level was raised by approximately 31% and approximately 55% in GW- and AG-treated samples, respectively, when compared to that of infection only (Fig. 6A, B, E, and F). Furthermore, AT1 protein level was increased by approximately 60, 85% and 35, 49.5% in infected and treated with either GW or AG, respectively, as compared to mock (Fig. 6A, C, E, and G). However, PPARγ level was decreased by approximately 26, 42% and 40, 73%, respectively, in infected and treated with either GW or AG as compared to mock (Fig. 6A, D, E, and H). Interestingly, the levels of JEV-NS3 and AT1 proteins were decreased by approximately 44% and 35.5%, respectively, in TM-treated samples as compared to infection only (Fig. 6I through K). Surprisingly, PPARγ expression was increased by approximately 84.5% as compared to infection only (Fig. 6I and L).

To assess the effect of the inhibitors on the cells in the absence of viral infection, the BHK-21 cells were treated with TM (25 and 50 µM), and the cell pellet was harvested after 24 hours. There was no significant change in the expression levels of AT1 and PPARγ when compared to the mock cells (Fig. S3A through C). Similarly, the uninfected BHK-21 cells were treated with GW (10 and 20 µM) and AG (10 and 20 µM) for 24 hours. Interestingly, the AT1 expression level was increased significantly in AG-treated cells, whereas it remained unaltered in GW-treated cells as that of the untreated mock control (Fig. S4A, B, D, and E). Furthermore, the PPARγ level remained unchanged in both AG- and GW-treated cells as compared to mock cells (Fig. S4A, C, D, and F).

**Fig 7:**
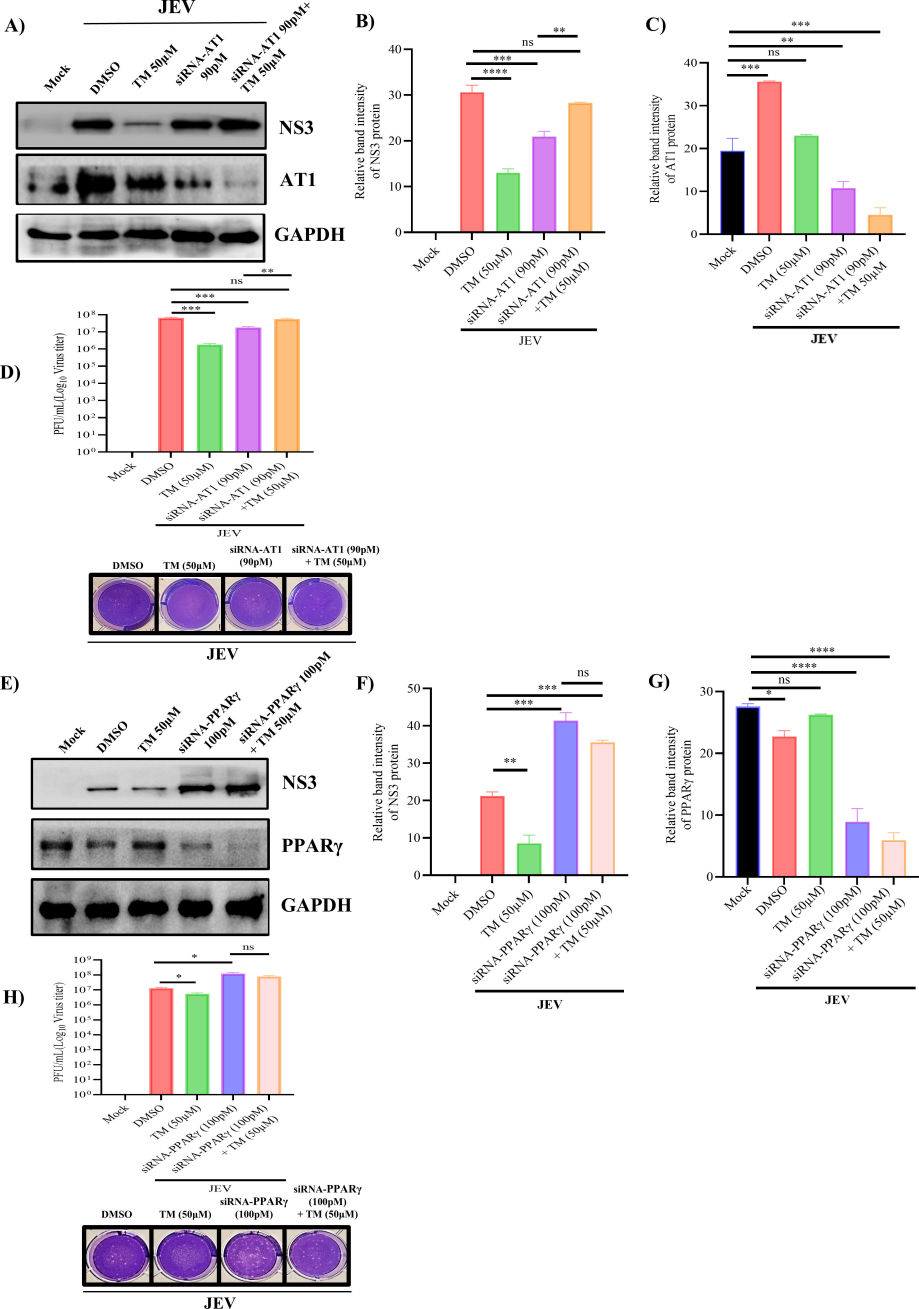
Knockdown of AT1 and PPARγ to understand the mode of action of TM: Huh7 cells were transfected with siRNA for AT1 and PPARγ. Next, the cells were infected with JEV, and TM was added post-infection. (**A and E**) Western blot image showing JEV-NS3 AT1 and PPAR**γ** protein levels in different treatment conditions. (**B, C, F, and G**) Bar diagrams representing relative band intensities of NS3, AT1, and PPAR**γ** proteins, respectively. (**D through H**) Bar diagrams showing viral titer as plaque-forming units (PFU/mL). The lower panel shows the images of the respective plaque assay plates. All the statistical analysis was carried out using the one-way ANOVA test. Data were representative of three independent experiments. *P ≤ 0.05; **P ≤ 0.01; ***P ≤ 0.001 and ****P ≤ 0.0001 were considered statistically significant.

Furthermore, to understand the specific role of AT1 and PPARγ in TM-mediated JEV abrogation, siRNA-based knockdown experiments of AT1/PPARγ were performed in Huh7 cells. The knockdown efficiency for siRNA against AT1 (90 pM) and PPARγ (100 pM) was found to be ~ 60% and 50%, respectively (Fig. S5A through D). Next, the siRNA-transfected cells (48 hours post-transfection, hpt) were infected with JEV, and cells as well as supernatants were collected at 24 hpi to assess the viral protein and viral titer. The siRNA-AT1-transfected and JEV-infected cells exhibited a decrease in the viral NS3 protein expression (33%) and in viral titer (75%) when compared to the infection control. However, there were no significant changes in JEV-NS3 level and viral titer when TM was added along with siRNA-AT1-transfected and -infected cells as compared to the infection control (Fig. 7A through D). Whereas in siRNA-PPARγ knockdown condition, the viral NS3 protein expression level and viral titer were increased significantly by 50% and 88%, respectively, when compared to the infection control (Fig. 7E through H). However, in the TM-treated condition, no significant reduction was observed in JEV-NS3 protein level and viral titer as compared to only siRNA PPARγ-transfected and -infected cells.

Taken together, the above findings suggest that the mechanism of action of TM against JEV is mediated through the AT1/PPARγ axis.

### TM restricts the inflammatory response induced by JEV infection via p-NF-κβ/COX-2/p-ERK pathways

**Fig 8:**
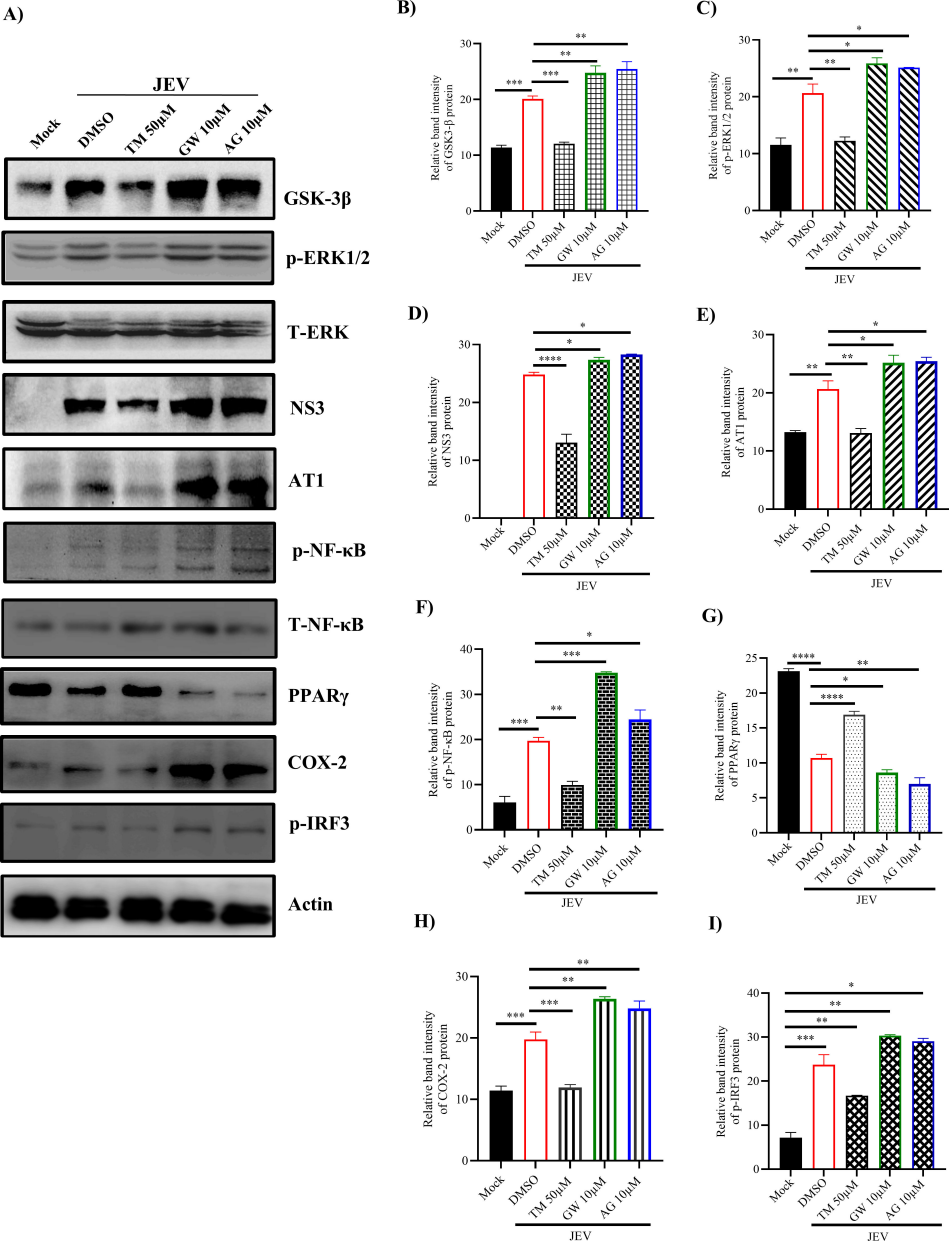
TM downregulates the inflammatory response induced by JEV infection via p-NF-κB /COX-2/p-ERK pathways: RAW 264.7 cells were pre-treated with GW (10 µM), AG (10 µM), and TM (50 µM) for 3 hours and then infected with JEV at an MOI of 5. The GW (10 µM), AG (10 µM), and TM (50 µM) were again administered post-infection, and cell pellets were collected after 24 hpi. (**A**) Western blot image showing expression profiles of GSK-3β, T-ERK, NS3, AT1, T-NF-κB, PPARγ and COX-2 proteins along with phosphorylated ERK1/2, NF-κB, and IRF-3 in JEV-infected and TM-, GW-, and AG-treated RAW 264.7 cells. Actin was used as a loading control. (**B–I**) Bar diagram indicates the relative band intensity of GSK-3β, p-ERK1/2, NS3, AT1, p-NF-κB, PPARγ, COX-2, and p-IRF3 proteins in different samples. The statistical analysis was done by the one-way ANOVA test. *P ≤ 0.05, **P ≤ 0.01, ***P ≤ 0.001, and ****P ≤ 0.0001 were considered statistically significant, *n* = 3.

As per Sumarno et al. 2020 (15), the neuroprotective action of TM was mediated through the involvement of p-NF-κβ/COX-2/p ERK (phospho-nuclear factor kappa-light-chain-enhancer of activated B cells/cyclooxygenase 2/phospho-extracellular signal-regulated kinases factors). Thus, to further understand the role of downstream players of the AT1/PPARγ pathways, JEV infection was carried out in immune cells such as RAW264.7 at an MOI of 5 (due to less susceptibility to JEV infection) (16). It was observed that expressions of GSK-3β (glycogen synthase kinase-3 beta), p-ERK1/2, and AT1 were upregulated by 50; 75; 70%; 58; 79; 82% and 35; 57; 55% (approx) in infected and GW- or AG-treated cells, respectively, as that of mock (Fig. 8A, B, and E). Whereas in the TM-treated sample, the expressions of GSK-3β, p-ERK, and AT1 were downregulated significantly by approximately 58.5%, 52%, and 55% (Fig. 8A, B, C, and E) when compared to infection only. The activation of p-NF-κB and COX-2, a prostaglandin maker enzyme involved in inflammation, was raised by 42.5; 70; 65% and 51; 83; 70% (approx) in infected and GW- or AG-treated and infected samples, respectively, as that of mock. Whereas the p-NF-κB and COX-2 protein levels were downregulated by 51% and 52% (approx), respectively, in TM-treated samples when compared to infection (Fig. 8A, F, and H). The PPARγ level was reduced by 54; 62; 64.5% (approx) in infected and GW- or AG-treated samples as compared to mock. Furthermore, in TM-treated samples, it was increased significantly by 32% as compared to infection control (Fig. 8A and G). Similarly, the JEV-NS3 protein was also reduced significantly by 44% when compared to infection only, whereas it was upregulated in AG/GW- (10% and 14%) treated RAW264.7 cells during viral infection (Fig. 8A and D). Furthermore, there was a 39% reduction in the level of inflammatory cytokine [p-IRF3 [phospho-interferon regulatory factor 3]) in TM-treated conditions as compared to infection control (Fig. 8A1). Taken together, the data indicate that TM efficiently reduces JEV-induced inflammation by downregulating p-IRF-3, COX-2, and p-NF-κB.

### JEV infection was reduced by TM in the mouse model

**Fig 9:**
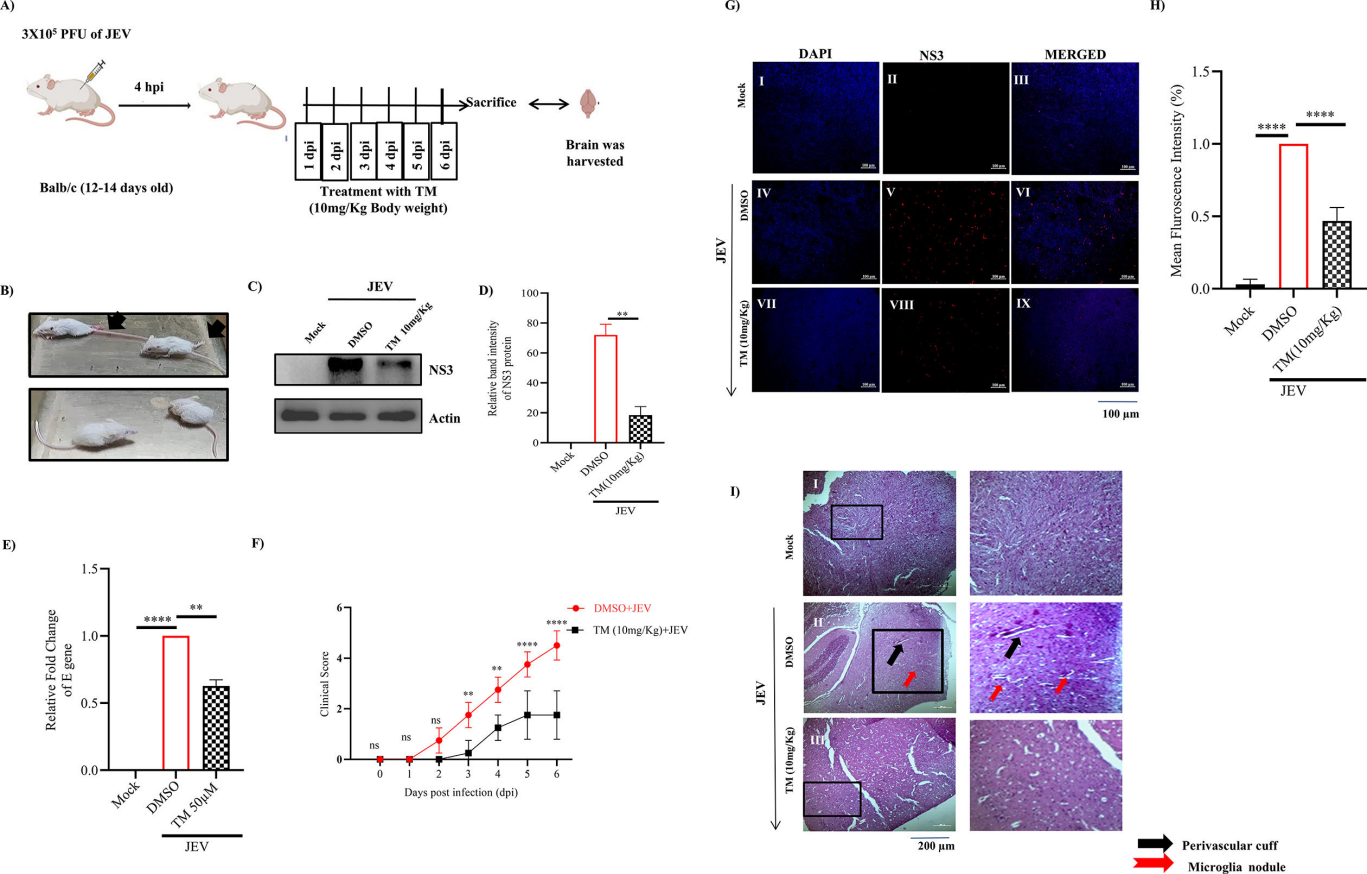
TM reduces JEV infection in mice model: The Balb/c (12–14 days old) mice were injected subcutaneously with 3 × 10^5^ PFU of JEV, and TM (10 mg/Kg) was given orally at an interval of 24 hours up to 6 days. Mice were sacrificed on the 7th day post-infection; sera and brain tissue were collected for further downstream processing. (**A**) Schematic diagram representing experimental workflow in the mouse model. (**B**) Image representing JEV-infected and drug-treated mice and arrow indicating hind limb paralysis in infected mice at 6th dpi. (**C**) Western blot image depicting the JEV-NS3 protein in brain tissue samples of mock, infected, and treated mice. (**D**) Bar diagram indicating relative band intensity of JEV-NS3 protein in different samples. (**E**) Bar diagram showing relative fold change of viral E gene expression in various samples. (**F**) Graph showing the clinical scores of the disease symptoms of JEV-infected and -treated mice, which were under observation from 1 dpi to 6 dpi (*n* = 4). (**G**) Confocal images representing mock, JEV-infected, and infected along with treated brain tissue samples stained for the JEV-NS3 protein (scale Bar 100 µM). (**H**) Bar diagram showing mean fluorescence intensity in different samples. (**I**) Image showing H&E-stained mock, infected, and drug-treated brain tissue samples, black arrow indicating perivascular cuffs and red arrow indicating microglia nodules. Scale bar 200 µM. Data from three independent experiments are shown as mean ± SD.

Next, the anti-JEV activity of TM was evaluated in Balb/c mice (12–14 days old). The mice were infected with JEV, and a 10 mg/Kg dose of TM was administered orally at an interval of 24 hours up to 6 dpi (Fig. 9A). The JEV-infected mice showed a rise in body temperature, ruffled fur, hunch back, slow movement, and hind limb paralysis, whereas TM-treated animals showed no such symptoms (Fig. 9B). Furthermore, the expression of the NS3 protein as well as JEV-E gene was reduced drastically (>60% and 40%, respectively) in TM-treated mice brain as that of infected one (Fig. 9C, D, and E). The clinical score graph showed lessening of symptoms in TM-treated mice (Fig. 9F), and immunohistochemistry analysis revealed approximately 54% decrease in the JEV-NS3 protein level in the drug-treated mice brain when compared to infection only (Fig. 9G and H). Interestingly, JEV-infected brain tissue sections showed an increased number of perivascular cuffs and microglia nodules, which were found to be reduced significantly in TM-treated sections (Fig. 9I). Thus, the above findings indicate that TM can reduce JEV infection and protect mice efficiently.

## DISCUSSION

In the Asia-Western Pacific region, JEV is the most prominent cause of encephalitis, which is associated with high mortality or neuropsychiatric sequelae (4). While the vaccines have failed to eradicate JEV, efforts have not been successful in developing a clinically effective antiviral strategy. Considering the proinflammatory nature of the AT1 axis in inducing encephalitis, ARBs can help manage JEV infection. Because of the relatively higher ability to cross the BBB, TM can be prioritized among the ARBs. It is also a partial agonist of PPARγ, which has been reported to be downregulated during viral encephalitis (11). Other than mediating encephalitis, the AT1/PPARγ axes are also critical host factors for the replication of several alphaviruses and flaviviruses. Considering TM’s ability to modulate these axes, there is great interest in investigating its efficacy against JEV infection.

In the current study, TM was found to be non-cytotoxic to BHK-21 cells until 160 µM and efficiently inhibited JEV infection (MOI of 0.1) in the BHK-21 cell line with 50 µM (90%) and 75 µM (95%) concentrations following post-infection. This was also associated with a significant reduction in viral RNA (50% at 50 µM; 75% at 75 µM) and protein levels (52% at 50 µM; 70% at 75 µM). This anti-JEV property was also supported by reduced expressions of JEV-E, iNOS, and Cas3 genes in physiologically relevant cell lines, including macrophages (RAW264.7) and neuronal (SH-SY5Y) and human (HEK293 and Huh7) cells. Interestingly, TM was able to reduce the viral load in pre-, co-, and post-treatment conditions. Interestingly, viral titer was also reduced significantly (30%) when the virus particles were incubated with TM. Thus, some virus inactivation activity of TM cannot be ruled out. TM is known to be an AT1 blocker and a partial PPARγ agonist. So, to understand the involvement of these axes in mediating TM’s antiviral action against JEV, agonists of AT1R (AG) and antagonists of PPARγ (GW) were used. Viral titer was enhanced (~ 80% and 90%) in the presence of GW/AG, suggesting that TM could abrogate viral infection through the AT1/PPARγ axis. The AT1 protein level was upregulated (~ 60, 85 and 35, 49.5%), whereas the PPARγ level was downregulated (~ 26, 42 and 40, 73%) during infection and with GW- or AG-treated conditions. Interestingly, the AT1 (~ 35.5%) and PPARγ (~ 84.5%) levels were reversed in the presence of TM. In addition, TM showed a significant reduction in the inflammatory responses by downregulating p-IRF-3, COX-2, and p-NF-κB. Further evaluation in the Balb/c mice model revealed that TM can remarkably reduce the disease score, viral protein level, and histological changes in the brain tissue of JEV-infected mice.

TM showed a dose-dependent reduction in viral titer, with about 95% abrogation of virus particle formation, with 75 µM concentration when administered post-JEV infection (0.1 MOI). A proportionate decrease in viral RNA and JEV-NS3 protein levels was also observed. Furthermore, the CC_50_ of TM was found to be >350 µM (BHK-21 cells). A similar CC_50_ has been reported for TM in Vero and RAW264.7 cells (12). With an IC_50_ of 24.68 µM, the selectivity index of TM was estimated to be >14.18 (Fig. 2). This selectivity index and the fact that the clinical safety profile of TM is well established suggest its potential for repurposing. In further support of this, the effect of TM was demonstrated in physiologically relevant cell lines, including macrophages (RAW264.7), human (HEK293 and Huh7), and neuronal (SH-SY5Y) cells. A significant reduction was observed in the expression of JEV-E in a dose-dependent manner. Moreover, TM showed substantial reductions in INF-β gene expression and iNOS levels in RAW 264.7 cells. Since INF-β (17) and iNOS (18) are markers of neuroinflammation, this also indicates the neuroprotective potential of TM, which is necessary for the management of JE (19). Also, Cas3 genes were significantly reduced by TM in neuronal cell lines (SH-SY5Y), indicating decreased apoptosis. Taken together, these findings suggest the potential of TM to manage JE.

Although TM reduced the viral load in pre- and co-treatment conditions, a significant reduction of viral titer upon post-treatment indicates some host-mediated actions. Nonetheless, it also showed a 30% abrogation of virus particles following incubation with TM, which suggests some direct antiviral effects. However, no specific virus targets can be identified at this point. TM has earlier been reported to directly affect the protease activity for CHIKV-nsP2 (20). However, the same cannot be said about JEV, and this needs further investigation. Hence, TM can be said to have predominantly host-mediated antiviral properties against JEV. Nevertheless, considering its significant reduction in pre-treatment conditions, the involvement of some viral targets cannot be ruled out.

To understand the mechanisms of action of TM, the primary mediator of its pharmacodynamics, AT1 protein, and PPARγ were monitored. The AT1 protein expression was enhanced in infected cells, whereas PPARγ expression was downregulated. This suggests the involvement of both these axes in JEV infection. Furthermore, TM was not able to reduce the viral infection significantly upon siRNA-based knockdown experiments. Entry and replication of flaviviruses are reported to be modulated by AT1 activation (21). For example, C-type lectin on dendritic cells called dendritic cell intercellular adhesion molecule 3-grabbing nonintegrin (DC-SIGN) and glucose-regulated protein-78 (GRP78), which play a critical role in JEV entry as well as replication (22, 23), are reported to be upregulated with AT1 activation (21) during infection of JEV as well as some other flaviviruses, including DENV. Hence, the upregulation of the AT1 protein may have contributed to the entry and replication of JEV infection. Although there is no specific data about the involvement of PPARγ in JEV entry or replication, it has pleiotropic roles in viral infections and inflammations in the brain (11). Thus, the reduction in PPARγ expression may have contributed to the rise in infection in multiple ways, which need further investigation. In the present study, the AT1 level was downregulated, and the PPARγ level was upregulated upon TM treatment. Altogether, the above findings suggest the pivotal role of AT1 and PPARγ in the inhibitory action of TM against JEV, which can be further explored for therapeutic intervention for multiple viral infections.

**Fig 10:**
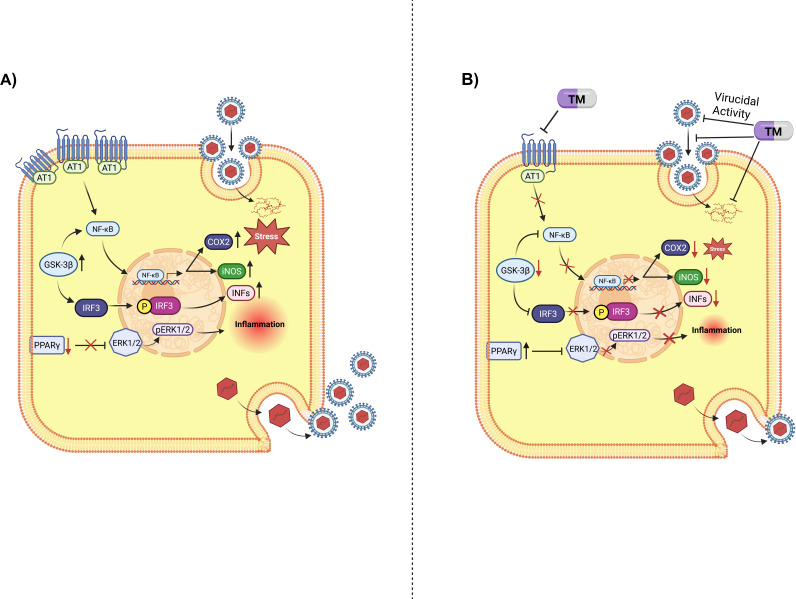
Diagrammatic representation of the proposed working model of TM: RAW264.7 cells were infected with JEV (MOI 5), and 24 hpi cells were harvested. (**A**) Image showing JEV infection alters expressions of AT1 (increased), PPARγ (reduced) and other downstream key players such as p-ERK1/2, p-NF-κB, p-IRF3, GSK-3β, and COX-2. (**B**) Image representing TM treatment leads to decreased AT1 and increased PPARγ expressions which further leads to decreased phosphorylation of ERK1/2, NF-κB, and IRF3 and also downregulates the expression of GSK-3β as well as COX-2. Also, TM interferes with the JEV life cycle, entry, and also possesses virucidal activity, resulting in decreased viral load. This explains the reduction in oxidative stress and inflammation, preventing cell injury and death.

To further support this mode of action, some downstream players of AT1 and PPARγ were explored. Key downstream players of the AT1/PPARγ pathway were analyzed, demonstrating the reduction of inflammatory markers such as p-IRF3, GSK-3β, p-ERK1/2, p-NF-κB, and COX-2. The gene expression levels of GSK-3β, COX-2, and p-NF-κB proteins are reported to be higher in JEV, and their reductions have been associated with a decrease in JEV infection in human microglia (CHME3) and human neuroblastoma cells (SH-SY5Y) (24). Thus, it can be proposed that in JEV infection, AT1 was upregulated, which, in turn, activates GSK-3β. At the same time, viral infection reduces PPARγ, which triggers the increased production of transcription factors such as p-IRF3, p-NF-κB, and pro-inflammatory markers COX-2. Finally, it results in increased viral infection, cell injury, and death (Fig. 10A). On the other hand, the presence of TM leads to a reduction of AT1, pro-inflammatory cytokines, GSK-3β, and an increase in PPARγ, resulting in abrogation of viral infection and inflammation (Fig. 10B).

Furthermore, it has been reported by Rong Jiang et al. (25) that p-ERK1/2 level is upregulated in JEV infection. Moreover, JEV infection leads to the overproduction of ROS and activation of nuclear factor kappa B (NF-κB) and other signaling pathways, such as ASK1-ERK/p38 (apoptosis signal**-**regulating kinase 1) mitogen-activated protein kinase (MAPK) (26), leading to inflammation. Also, the AT1 axis mediates inflammation through the NF-κB/cytokine pathway (27). Besides this host-mediated antiviral effect, TM directly impedes JEV infectivity. Taken together, TM inhibits viral infection directly and indirectly by modulating host factors and managing the JEV-induced inflammation.

The *in vitro intervention* was correlated with *in vivo* studies. With once-a-day oral administration of TM (10 mg/kg) for 6 days, the JEV NS3 protein expression was reduced drastically (approximately 60%) in the brain of the Balb/c mice, suggesting a significant reduction in JEV infection of the brain. The reduction in encephalitis was evident from a significant decrease in perivascular cuffs and microglia nodules. Since encephalitis and neurological sequelae are the primary reasons for mortality and morbidity, these findings demonstrate the efficacy of TM in the management of JE. Previous reports suggest the high doses of drugs NTZ (50 mg/kg/day, 75 mg/kg/day, and 100 mg/kg/day) and AV1004 (35 mg/kg) for 25 and 23 days to decrease JEV load, respectively (28, 29). Similarly, a report demonstrated that a 15 mg/kg twice daily berberine dose was given to 3- to 4-week-old BALB/c mice for up to 15 days to reduce JEV infection (30). Compared to these, TM was effective even after a shorter duration (6 days) of treatment. TM is widely used for prolonged therapy of hypertension at a dose of 40 mg/day or higher. Thus, the effectiveness of TM at the equivalent of this human dose in mice (10 mg/kg) suggests the possibility of its acceptability while repurposing. This is also helpful because, at these lower doses, TM is relatively safe in normotensive individuals (31). However, for effective repurposing, the dose regimen needs further optimization. The oral bioavailability of TM is dose-dependent (40% at 40 mg/day and 58% at 160 mg/day) (32). Since increasing doses for normotensive JEV patients may have adverse impacts, it is necessary to improve bioavailability at lower doses for repurposing applications. Also, morbidity and mortality of JEV are primarily higher in children and young adults; hence, the safety profile of TM in these groups needs to be evaluated. Alternatively, selective delivery of TM to the brain needs to be designed for effective repurposing against JEV.

### Conclusion

Both *in vitro* and *in vivo* findings show the ability of TM to reduce JEV infection. The AT1 and PPARγ axes, known to mediate encephalitis, were also found to be involved in JEV infection. Besides modulating these axes, TM may also interfere with some antiviral targets for its anti-JEV properties. Furthermore, TM can potentially manage JE by reducing the downstream markers of these axes that mediate inflammation. The preclinical efficacy at a human equivalent dose and with a relatively shorter duration of exposure suggests its suitability for further repurposing.

## MATERIALS AND METHODS

### Cells and viruses

The BHK-21, HEK293, Huh7, and RAW264.7 cells were procured from the National Centre for Cell Sciences (NCCS); Pune, India, whereas the PS (Porcine Stable Kidney) and SH-SY5Y cell lines were a kind gift from Dr A. Basu, (NBRC; Gurgaon, India) and Dr G. H. Syed (Institute of Life Sciences, Odisha), respectively. The BHK-21, HEK293, Huh7, and PS cells were maintained in Dulbecco’s modified Eagle’s medium (DMEM; Himedia, India), and RAW264.7 cells were maintained in Roswell Park Memorial Institute media (RPMI 1640, Gibco Gluta MAX; Invitrogen, Cal, US), respectively, along with 10% fetal bovine serum (Gibco, Invitrogen), gentamycin and penicillin-streptomycin (Sigma, USA). The SH-SY5Y (neuronal cells) was maintained in DMEM/F-12 media (Gibco). All the cells were kept in a 5% CO_2_ incubator at 37°C. The GP78 strain (accession No. AF075723) of JEV was a kind gift from Dr A. Basu (NBRC; Gurgaon, India).

### Antibodies and compounds

The JEV-NS3 (Non-structural protein-3) and actin antibodies were obtained from GeneTex (Cat. No. GTX125868) and Sigma-Aldrich (MO, US), respectively. The PPARγ, T-ERK1/2, p-ERK1/2, p-NF-κB, T-NF-κB, COX-2, GSK-3β, and p-IRF3 were procured from Cell Signaling Technology (CST) (MA, US). The AT1 antibody was obtained from Santa Cruz Biotech (TX, US). The [Val5]-angiotensin II acetate salt hydrate (AG) is an ANG II analog having valine instead of isoleucine in an ANG II octapeptide at the fifth amino acid position (Sigma-Aldrich, MO, US), and GW9662 (GW) is an irreversible PPARγ antagonist (Sigma-Aldrich, MO, US). GAPDH (glyceraldehyde-3-phosphate dehydrogenase) was procured from Imgenex India Pvt Ltd. Telmisartan (TM) was a kind gift from Glenmark Life Sciences Ltd., Ankleshwar, Gujarat, India.

### Virus infection

Cells were seeded onto the plate with 70%–80% confluency. The JEV infection was carried out at 0.1 MOI in BHK-21, HEK293, Huh7, and SH-SY5Y cells, whereas at an MOI of 5 in RAW264.7 cells in serum-free media (SFM) with shaking at an interval of every 15 min up to 90 min as per references (33–35). The cells were washed twice with 1× PBS, and complete media was then added along with the TM at varying concentrations post-infection. The cells were incubated at 37°C with 5% CO_2_ till 24 hours post-infection (hpi). The cell culture supernatant and pellets were harvested at 24 hpi for further downstream processing.

### Plaque assay

The plaque assay was performed as described earlier by De et al. (12). Briefly, the cell supernatant from mock, infected, and treated samples was collected and serially diluted in SFM. The diluted samples were added onto a monolayer of PS cells, and infection was carried out as described before. After infection, the cells were overlaid with carboxymethyl cellulose (Sigma-Aldrich, MO, US). The plates were incubated at 37°C with 5% CO_2_ for 6–7 days until clear plaques were visible. The cells were then fixed with 8% formaldehyde (Sigma-Aldrich, St. Louis, MO) and stained with 0.1% crystal violet (Sigma-Aldrich, St. Louis, MO). The number of plaques was calculated and presented as plaque-forming units per milliliter (PFU/mL).

### Cytotoxicity assay

The MTT assay was carried out using the EZcountTM MTT cell assay kit (Himedia, Mumbai, India) as per the manufacturer’s protocol. Approximately 10,000 BHK-21 and Huh7 cells were seeded in 96-well plates 24 hrs prior to infection. The BHK-21 and Huh7 cells were then treated with different concentrations of TM (5, 10, 20, 25, 40, 50, 75, 80, 100, 160, 200, 250, 350, and 500 µM) for 24 and 48 hours, respectively, along with a reagent control. However, cytotoxicity of GW (1, 5, 10, and 20 µM) or AG (10, 20, 30, and 40 µM) was also determined in BHK-21 cells (24 hours). The absorbance was taken at 570 nm. The percentage of metabolically active cells was compared with the control cells to determine cellular cytotoxicity 50 (CC_50_) as mentioned previously by De at al (12).

### Western blot

The Western blot analysis was done as described previously by Chatterjee et al. (36). In short, the mock, infected, and treated samples were harvested and lysed with RIPA lysis buffer. Similarly, mock, infected, and treated brain tissue was snap-frozen and lysed by the homogenizer in RIPA buffer. The equivalent concentration of each sample was loaded and run on a 10% SDS-polyacrylamide gel. The proteins from cell culture supernatant and brain tissue samples were transferred onto polyvinylidene difluoride (PVDF) membrane and probed with primary antibodies JEV-NS3, T-ERK1/2, p-ERK1/2, p-NF-κB, T-NF-κB, COX-2, GSK-3β, and p-IRF3, in 1:1,000 dilutions, AT1 (1:250), and PPARγ (1:250) for overnight. Furthermore, the brain tissue sample was probed with JEV NS3 (1:1,000) antibody. Actin (1:500) and GAPDH (1:4,000) were used as internal controls. The detection was done by corresponding secondary antibodies tagged with horseradish peroxidase (HRP). The blots were imaged by Chemidoc (BioRad), and the protein band was quantified using the Image J software (*n* = 3).

### Confocal microscopy

The immunofluorescence assay was performed as described previously by Chatterjee et al. (36). The BHK-21 cells were seeded onto coverslips in a six-well plate with 50% confluency. The cells were infected as described above. At 24 hpi, the cells were fixed with 4% paraformaldehyde and incubated with primary antibody JEV-NS3 (1:2,000) for 45 min. After washing the cells with 1× PBS, secondary antibody 594 anti-rabbit Alexa Fluor (Invitrogen) was added for 45 min. The cells were mounted with antifade reagent (Invitrogen) to avoid photobleaching. The images were acquired using the Leica TCS SP5 confocal microscope (Leica Microsystems, Heidelberg, Germany) with a 10× objective.

### Treatment of TM pre-infection, co-infection, and post-infection

The pre-, during, and post-assay were performed as described earlier by De et al. (12). In brief, BHK-21 cells were pretreated with TM (50 µM) for 3 hours, and later on, infection was carried out. In the during condition, the drug was given along with the virus, and then the infection was carried out for 1.5 hours. Furthermore, in the post-treatment, the drug was added after the virus infection. The supernatant from all the above-mentioned three conditions was collected at 24 hpi, and the viral titer was determined by plaque assay.

### Virucidal assay

The virucidal activity of TM was determined as mentioned previously by Kitidee et al. (37). In brief, the virus (0.1 MOI) was incubated with 50 µM TM for 1 hour in serum-free media at 37°C in a 5% CO_2_ incubator. The plaque assay was performed with the untreated and drug-treated inocula to determine the viral titer.

### Treatment with compounds (GW or AG)

The BHK-21 cells were pre-treated for 3 hours with increasing concentrations of GW (1, 5, 10, 20, and 40 µM) or AG (10, 20, 30, and 40 µM) as described previously by De et al. (12). Later on, infection with JEV (MOI 0.1) was given as mentioned above. Post-infection, again, GW or AG was administered with the above-stated concentrations, and samples were collected at 24 hpi.

### Flow cytometry

The flow cytometry analysis was done as described earlier by Sanjai Kumar et al. (38). In brief, mock, infected, and infected along with TM-treated cells were harvested at 24 hpi. These cells were fixed with 4% paraformaldehyde followed by resuspension in FACS buffer (1× PBS, 1% BSA, 0.01% NaN_3_; Sigma-Aldrich, St. Louis, MO). The cells were treated with permeabilization buffer (1× PBS, 0.5% BSA, 0.1% Saponin, and 0.01% NaN_3_), followed by blocking with 1% BSA at room temperature for 30 min. The primary antibody anti-rabbit JEV NS3 (1:1,000) was added for 45 min. Next, the cells were washed with permeabilization buffer, followed by the addition of fluorescent-labeled secondary Alexa Fluor (AF) 594-conjugated anti-rabbit antibody (Invitrogen, US antibody). Approximately 10,000 cells were acquired by the LSR-Fortessa Flow cytometer (BD Biosciences, CA, US) for each sample and analyzed by the FlowJo V10.7.1 (BD Biosciences, CA, US).

### qRT-PCR

The viral and total RNA was extracted from supernatant, cells, and brain tissue sample using the QIAamp viral RNA mini kit (Qiagen, Hilden, Germany) and TRIzol (Sigma), respectively, as described earlier by Chatterjee et al. (39). The cDNA (PrimeScript 1st strand cDNA synthesis kit, Takara, Japan) was synthesized using equal volume of viral RNA and 1 µg of total RNA as per the manufacturer’s instructions. The qRT-PCR was performed using the JEV-E (envelope) (40), interferon-β (INF-β), iNOS (inducible nitric oxide synthase) (41), and Cas3 (Caspase3) (37) gene-specific primers (Supplementary Table No.2) using Mesagreen Sybr green master mix (Eurogentec) as per the manufacturer’s protocol. GAPDH was used as a control (Supplementary Table No. 1). The copy number per mL was calculated as described previously by Sanjai et al. (38), whereas the change in gene expression was calculated by the 2^−ΔΔ CT^ method.

### siRNA transfection

As previously described by Jichen Niu et al. and Jinchao Xinga et al. (35, 34), Huh7 cells were seeded in a six-well plate at a confluency of 60% and transfected with 90 and 100 pM of siRNA for AT1 and PPARγ (Santa Cruz Biotechnology, sc-29750 and sc-44220), respectively, for 48 hours using lipofectamine 3000 reagent (Invitrogen) as per the manufacturer’s instructions. After 48 hpt (hours post-transfection), cells were infected with JEV at an MOI of 0.1 as described earlier, and telmisartan (50 µM) was administered post-infection. The cells and supernatants were harvested at 24 hpi and processed by western blotting and plaque assay.

### Animal studies

The JEV infection was performed in 12- to 14-day-old Balb/c mice as described previously by Swarup et al. (42). In short, a lethal dose of 3 × 10^5^ PFU of JEV (GP78 strain) was injected subcutaneously. After 4 hours post-infection, the drug was given orally at a dose of 10 mg/Kg at an interval of 24 hours up to 6 days post-infection (dpi) to the treated mice group (*n* = 4). At 7th dpi, the mice were sacrificed. Brain tissue was collected and processed for Western blotting and qRT-PCR, respectively. The clinical score was recorded for each mouse depending upon the onset of symptoms on a daily basis (0, no symptoms; 1, ruffled fur; 2, hunch back; 3, slow movement; 4, hind limb paralysis; 5, death).

### Histopathological analysis

Histopathological analysis was carried out as described earlier by De et al. (12) with little modification in the protocol. For histopathological examinations, formalin-fixed tissue samples were dehydrated and embedded in paraffin wax, and serial paraffin sections of 5 mM were obtained. The cut sections were then heated at 54°C in a water bath and then dried at 37°C overnight (43). The sections were then stained with hematoxylin and eosin (H&E) (Himedia Cat no. S014-500ML and S007-500ML, respectively), and histopathological changes were visualized using a light microscope (Zeiss Vert.A1, Germany). Sections were also examined for the presence of JEV-NS3 protein using a specific antibody. Briefly, NS3 antibody (1:750) was added to the slides overnight at 4°C. After washing with 0.1% PBST thrice, slides were incubated with Alexa Fluor 594 (1:750; anti-rabbit; Invitrogen, MA, US) for 45 min−1 hour at room temperature in a dark and humidified chamber. The slides were washed thrice with 0.1% PBST and then mounted with a mounting reagent using DAPI (Invitrogen, MA, US).

### Statistical analysis

The statistical analyses were performed using the GraphPad Prism version 8.0.1 software. Data from three independent experiments are shown as mean ± SD. *P < 0.05, **P < 0.01, ***P < 0.001, *****P < 0.0001 were considered as statistically significant. For three groups, one-way analysis of variance (ANOVA) with Brown-Forsythe test was used, whereas for comparing two values, Student’s *t* Test was carried out. All the experiments were performed in triplicate.

## Data Availability

The data that support the findings of this study are available in the article.

## SUPPLEMENTAL MATERIAL

Supplemental figures and tables spectrum.03003-24-s0001.docx Fig. S1 to S5, and Table S1 and S2.

ASM does not own the copyrights to Supplemental Material that may be linked to, or accessed through, an article. The authors have granted ASM a non-exclusive, world-wide license to publish the Supplemental Material files. Please contact the corresponding author directly for reuse.
