# Supplementary material for: Telmisartan, an anti-hypertensive drug, impedes JEV infection, possibly via the AT1/PPARγ axis
Source: Microbiol Spectr. 2025 Aug 14;13(10):e03003-24. doi: 10.1128/spectrum.03003-24 (PMC12502669; doi:10.1128/spectrum.03003-24)

**Title: Telmisartan, an anti- hypertensive drug impedes JEV infection possibly via AT1/PPARγ axis**

**Authors:** Ankita Datey^a, b^, Sanchari Chatterjee^a, c^ **^#^**, Soumyajit Ghosh^a, d^ **^#^**, P Sanjai Kumar^a, e^, Saikat De^a, f^, Udvas Ghorai^a^, Debasish Hota^g^, Bharat Bhusan Subudhi^h, *^ and Soma Chattopadhyay^a, *^

Affiliation:

a) BRIC- Institute of Life Sciences, Bhubaneswar, Odisha, India.

b) School of Biotechnology, Kalinga Institute of Industrial Technology, Bhubaneswar, India.

c) Viral Mutation Section, HIV dynamics and Replication Program, Center for Cancer Research, National Cancer Institute at Frederick, MD, 21702, USA

d) Regional Centre for Biotechnology, Faridabad, India.

e) Division of Neonatology and Newborn Nursery, University of Wisconsin, Madison

f) Scripps Research, 10550 North Torrey Pines Road, La Jolla, CA 92037 (858) 784-1000

g) Department of Pharmacology, AIIMS, Bhubaneswar-751019, India.

h) School of Pharmaceutical Sciences, Siksha ‘O’ Anusandhan Deemed to be University, Sum Hospital Rd, Kalinganagar, Bhubaneswar, Odisha, India.

**#** Equal contributors

Email IDs: dateyankita@gmail.com

c.sanchari1712@gmail.com

soumyajitghosh12@gmail.com

udvasghorai@gmail.com

sanjai27112009@gmail.com

[ashirbadsaikatde@gmail.com](mailto:ashirbadsaikatde@gmail.com)

pharm_debasish@aiimsbhubaneswar.edu.in

* Corresponding authors,

Address of corresponding authors:

Dr. Soma Chattopadhyay (SC)

Infectious Disease Biology,

BRIC-Institute of Life Sciences (BRIC-ILS)

Nalco Square, Bhubaneswar, Odisha, India-751023

Phone No: 0091 674 2301476, ext 235; Fax No: 0091 674 2300728

E-mail: sochat.ils@gmail.com

And

Dr. Bharat Bhusan Subudhi (BBS),

Professor (Associate)

Drug Development and Analysis Lab., School of Pharmaceutical Sciences,

Siksha ‘O’ Anusandhan Deemed to be University, Kalinganagar, Bhubaneswar-751003,

Odisha, India

Phone No.: 9853945363

E-mail: bharatbhusans@gmail.com

**Fig. S1.** **TM efficiently reduces JEV load in physiologically relevant cell lines:** The RAW264.7 and SH-SY5Y cells were infected with JEV at a MOI of 5 and 0.1 respectively. After 24hpi cell pellets were harvested in TRIzol and total RNA was extracted. The qRT-PCR was performed with JEV-E (envelope) and Interferon beta (INF-β) genes specific primers. (A) Bar diagram representing the relative fold change of E gene in RAW264.7 cells (mock, infected and treated with different concentrations of TM). (B and C) Bar diagrams showing the relative fold change in INF-β and iNOS levels in mock, infected and treated RAW264.7 cells with varying concentrations of TM. (D and E) Bar diagram depicting reduction E and Cas3 gene expression in treated samples as compared to infection only in SH-SY5Y cells. (F, G, H and I) Bar graph representing reduction of E gene expression and viral titer following TM (50 and 75µM)) treatment in HEK293 and Huh7 cells respectively. (J) Bar diagram representing percentage viability of Huh7 cells treated with different concentrations of TM for 48hrs. The one-way ANOVA test was performed for statistical analysis *, *P* ≤ 0.05; **, *P* ≤ 0.01; ***, *P* ≤ 0.001; and ****, *P* ≤ 0.0001 were considered statistically significant, n=3.

**Fig. S2. Viral load abrogates with increasing concentrations of GW or AG:** BHK-21 cells were seeded onto coverslip and GW/AG were added pre and post infection. The infection was carried out at 0.1 MOI. The cells were fixed after 24hpi and incubated with JEV-NS3 (1:2000) primary antibody. (A and C) Confocal images showing number of JEV infected cells with increasing concentration of GW and AG respectively. (B and D) Bar diagram representing number of JEV-NS3 positive cells. (E) Bar diagram showing increase of viral RNA copy number/mL in 20 and 40µM of GW treated BHK-21 cells. (F) Bar graph showing increased viral titer in dose dependent GW treated conditions. Lower panel shows the plaque assay image for the same. The statistical analysis was done using the one-way ANNOVA. *, *P* ≤ 0.05; **, *P* ≤ 0.01; ***, *P* ≤ 0.001; were considered statistically significant whereas ns denotes non-significant.

**Fig. S3. AT1 and PPARγ expressions remain unchanged after TM treatment in mock cells:** BHK-21 cells were treated with TM (25 and 50µM) and after 24hpi cells were harvested, lysed with RIPA buffer. (A) Western blot image depicting expressions of AT1 and PPARγ proteins in TM treated mock cells. (B-C) Bar diagram showing relative band intensities of AT1 and PPARγ proteins. Actin was used as a loading control. The statistical analysis was done by the one-way ANOVA test, (ns=non-significant).

**Fig. S4. Expression profile of AT1 and PPARγ proteins in presence of AG and GW inhibitors:** BHK-21 cells were incubated with AG and GW inhibitors (10 and 20µM) and cell pellets were collected after 24 hpi. (A and D) Western blot image representing AT1 and PPARγ expression in AG and GW treated cells respectively. (B and E) Bar diagram showing relative band intensities of AT1 protein in AG and GW treated mock cells respectively. (C and F) Bar diagram indicating relative band intensities of PPARγ protein in AG and GW treated BHK-21 mock cells respectively. Actin was used as a loading control. The analysis was carried out using the graphpad prism tool by the one-way ANOVA test. *, *P* ≤ 0.05; **, *P* ≤ 0.01; were considered statistically significant, n=3.

**Fig. S5.** **Knocking down of AT1 and PPARγ in Huh7 cells:** Huh7 cells were transfected with 90 and 100 pM of siRNAs for AT1 and PPARγ respectively for 48 hrs. (A and C) Western blot images representing AT1 and PPAR γ protein expressions in mock and siRNA transfected cells respectively. (B and D) Bar graphs representing the relative band intensities of AT1 and PPARγ proteins respectively. The analysis was carried out using the graphpad prism tool by the one-way ANOVA test. **, *P* ≤ 0.01; ***, *P* ≤ 0.001 were considered statistically significant, n=3

| **S.No.** | **Gene Name** | **Sequence** |
| --- | --- | --- |
| 1. | Envelope (E) | FP:5’-TTGACAATCATGGCAAACGA-3’  RP:5’-CCCAACTTGCGCTGAATAAT-3’ |
| 2. | GAPDH | FP:5’-GAGTCAACGGATTTGGTCGT-3’  RP:5’-GACAAGCTTCCCGTTCTCAG-3 |
| 3. | iNOS | FP:5’-CGAGACGGATAGGCAGAGATTG-3’  RP:5’-CTCTTCAAGCACCTCCAGGAA-3’ |
| 4. | INF-β | FP: 5’-CCTCTCCATCAACTATAAGC-3’  RP:5’-CAACAATAGTCTCATTCCAC-3’ |
| 5. | Cas3 | FP:-5’-ACATGGCGTGTCATAAAATACC-3’  RP:-5’-CACAAAGCGACTGGATGAAC-3’ |

**Supplementary Table No.1: List of primer sequences**

| **Cell** | **CC_50_** | **IC_50_** | **S.I. (Selectivity Index)** |
| --- | --- | --- | --- |
| BHK-21 cells | 350μM | 24.68μM | 14.18 |

**Supplementary Table No.2: Selectivity Index of TM**


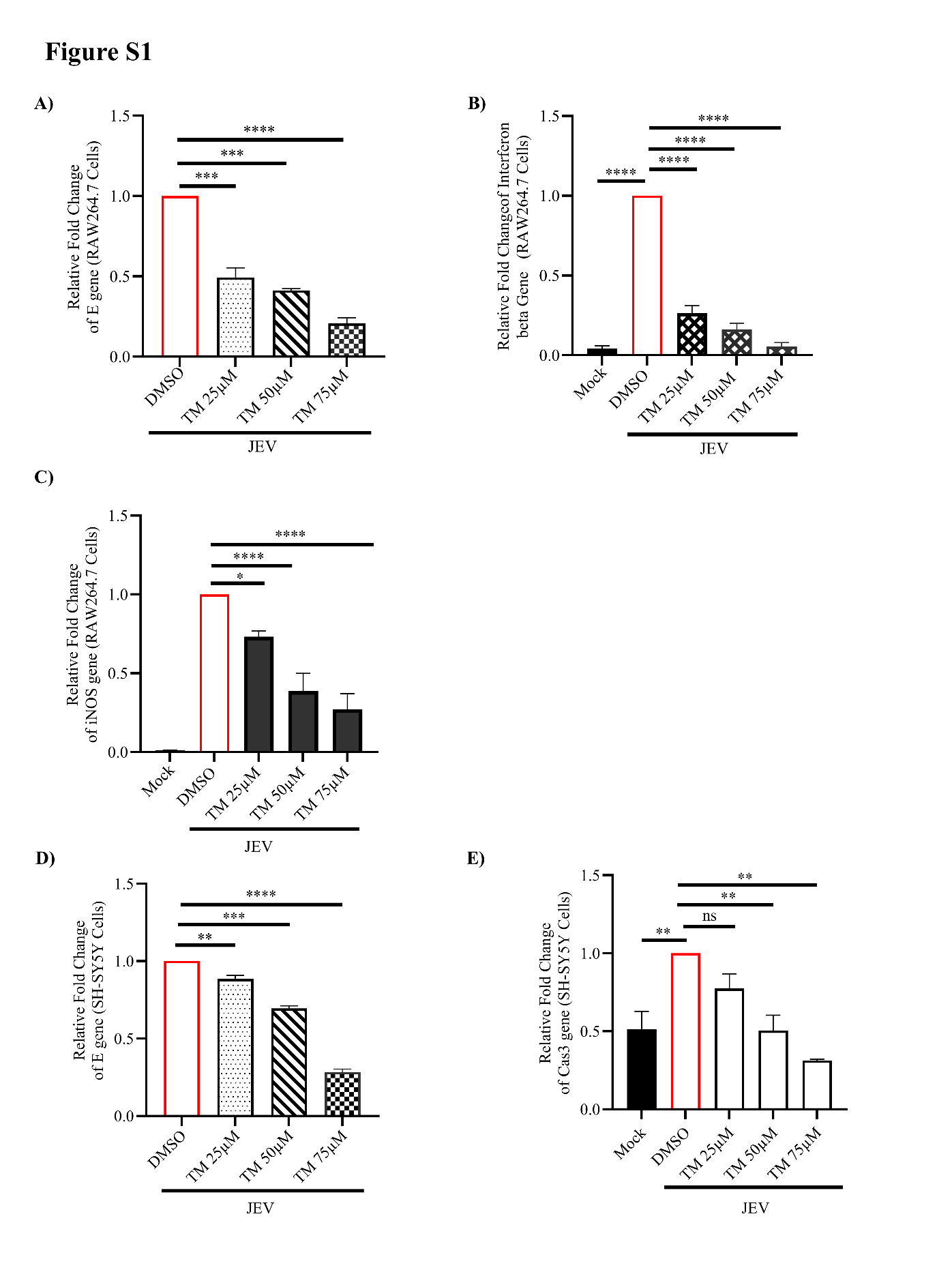


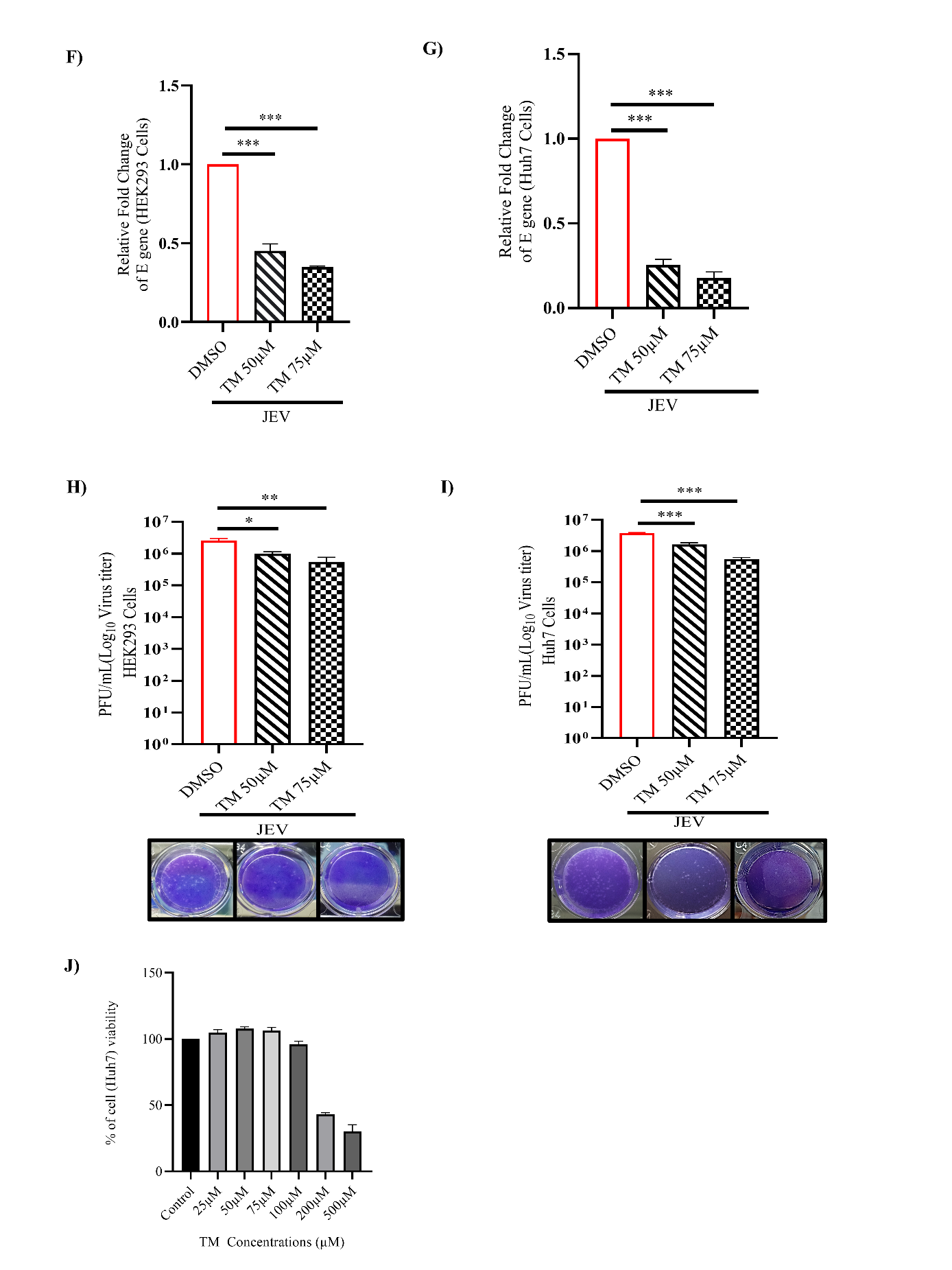

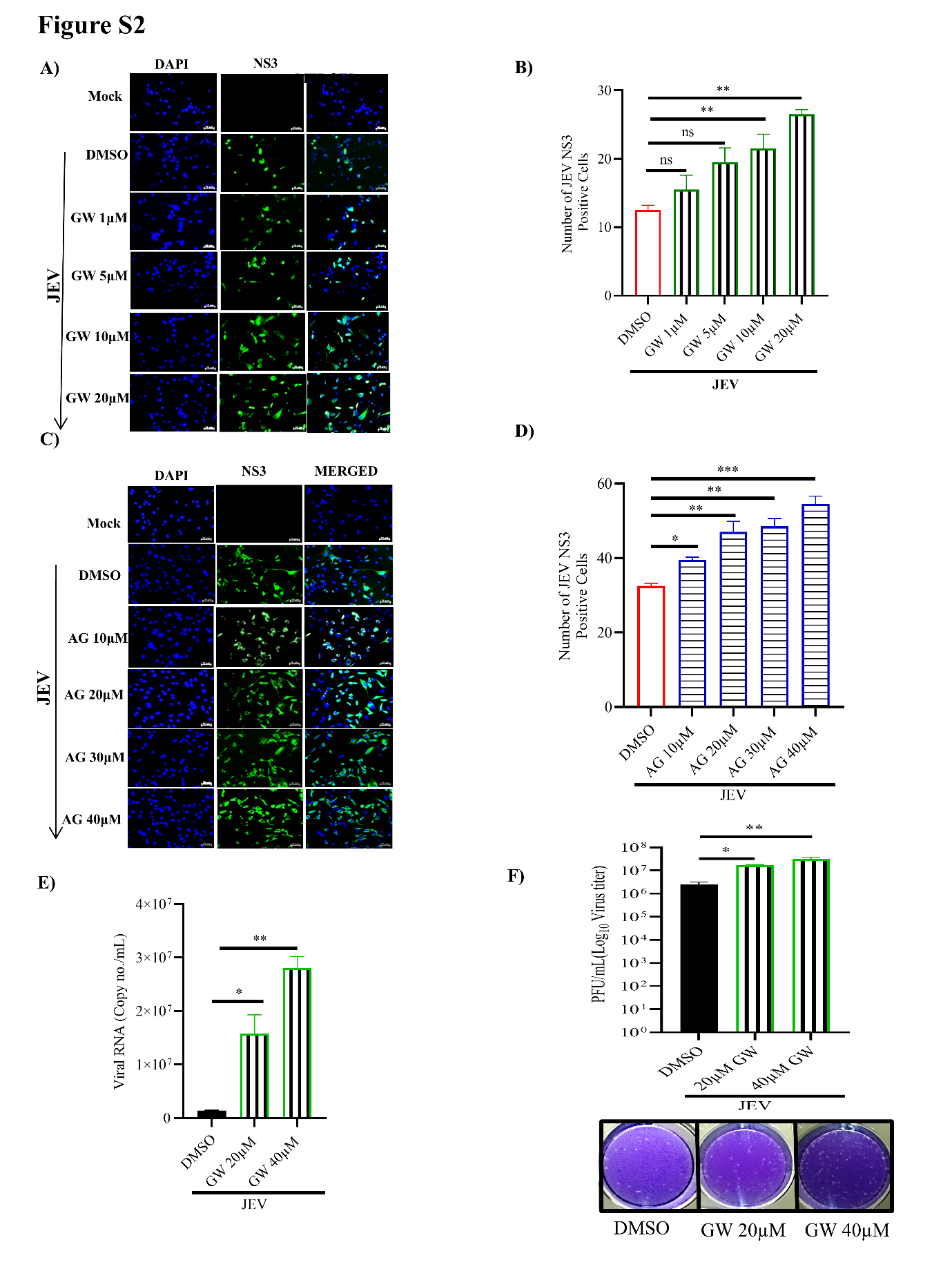


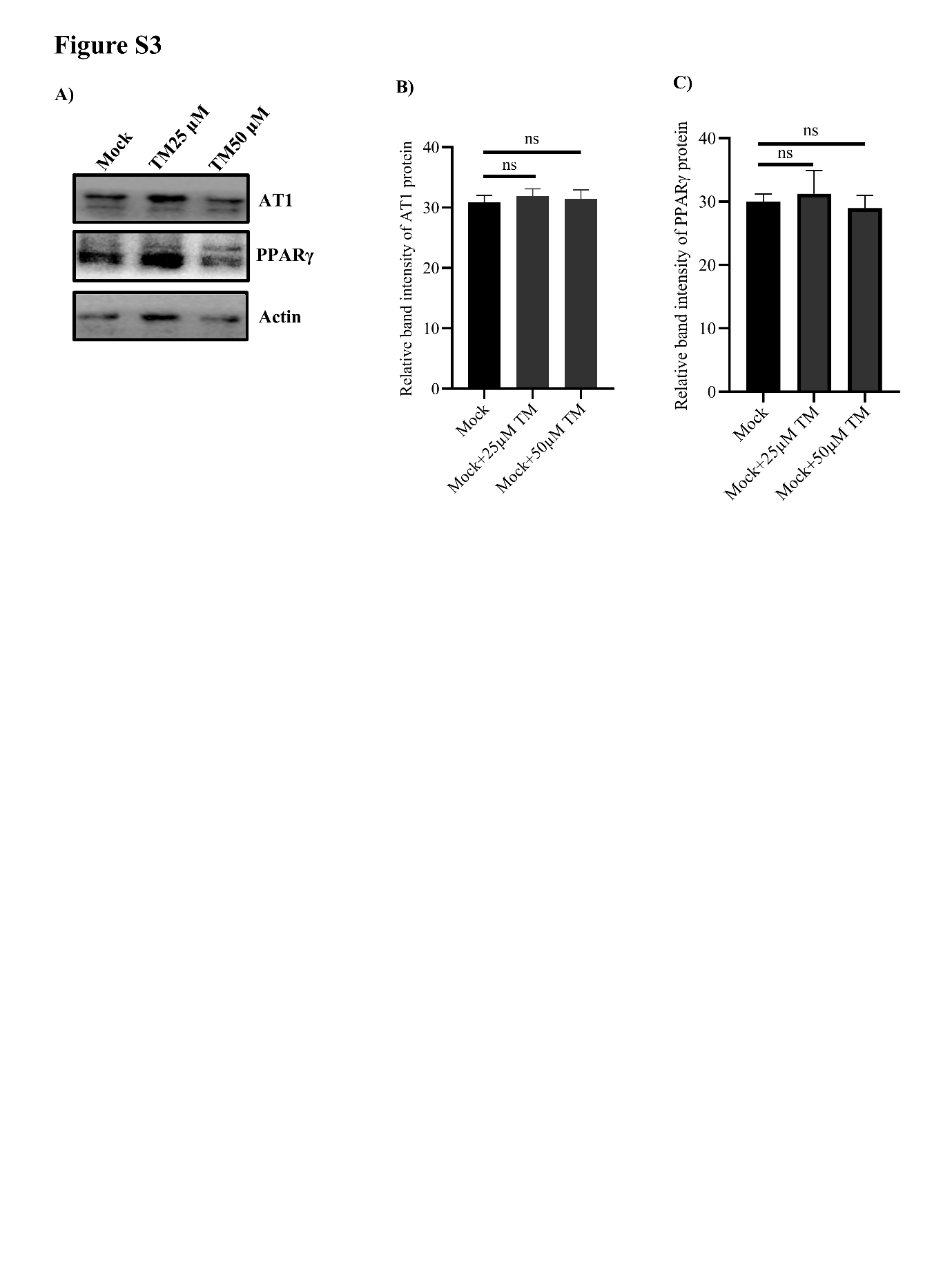

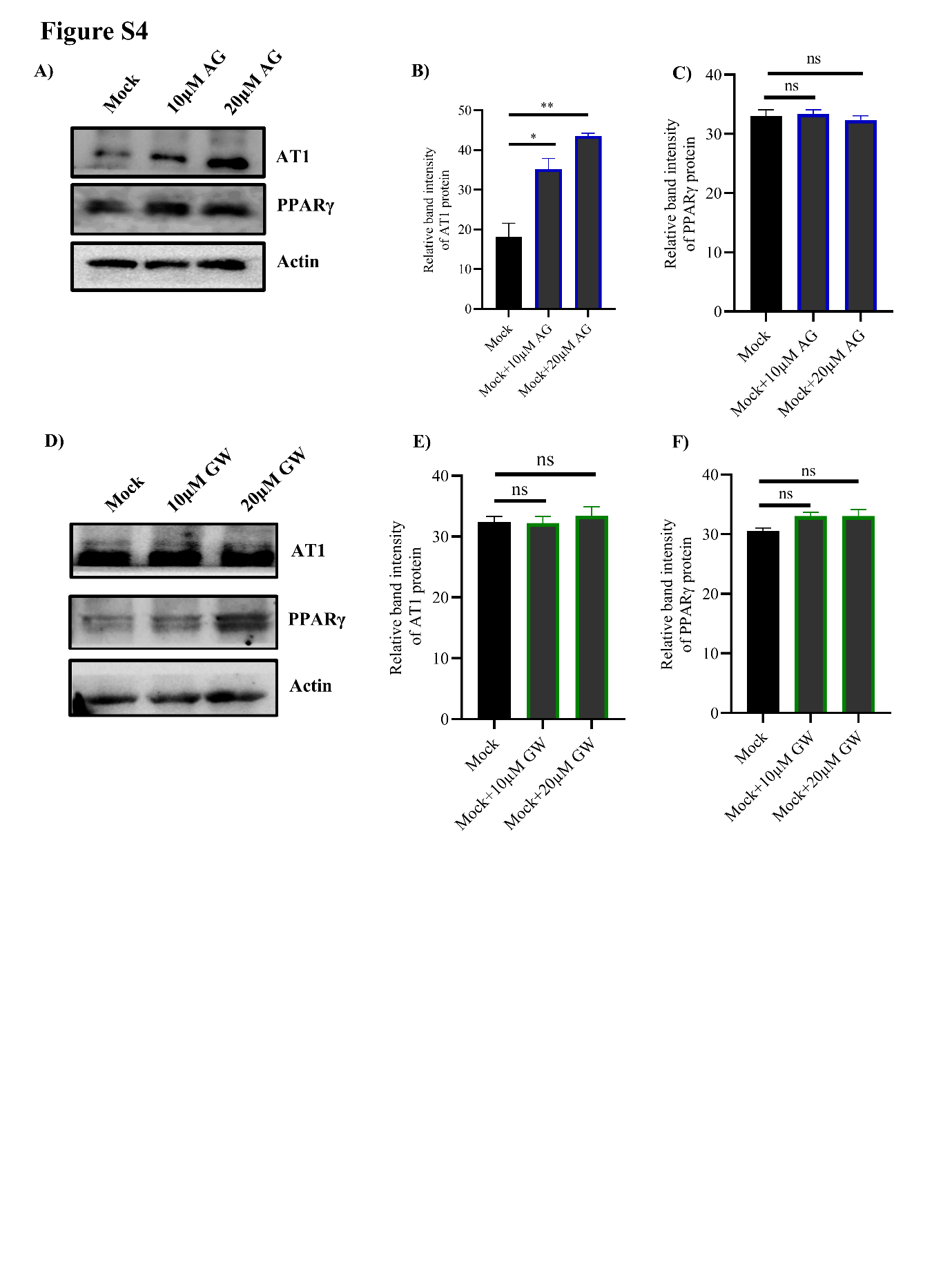

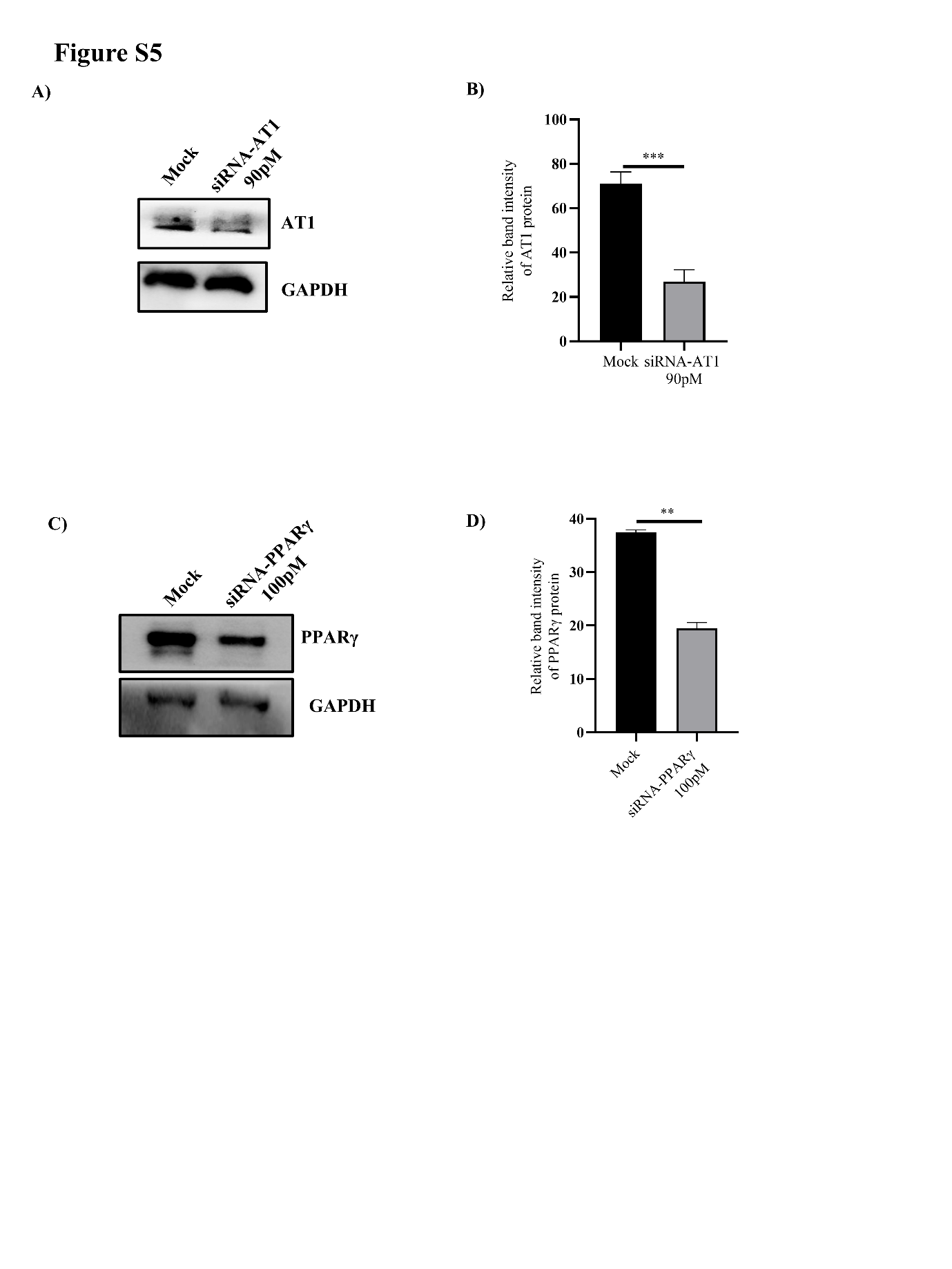

Supplement: Supplemental figures and tables — Fig. S1 to S5, and Table S1 and S2. [file spectrum.03003-24-s0001.docx]
